# A review of *Brucea javanica*: metabolites, pharmacology and clinical application

**DOI:** 10.3389/fphar.2023.1317620

**Published:** 2024-01-26

**Authors:** Jing Chen, Dongke Yu, Xinyu Li, Qichuan Deng, Hao Yang, Lu Chen, Lan Bai

**Affiliations:** ^1^ Department of Pharmacy, Personalized Drug Therapy Key Laboratory of Sichuan Province, Sichuan Provincial People’s Hospital, School of Medicine, University of Electronic Science and Technology of China, Chengdu, China; ^2^ The State Key Laboratory of Southwestern Chinese Medicine Resources, Department of Pharmacy, Chengdu University of Traditional Chinese Medicine, Chengdu, China; ^3^ Department of Pharmacy, Guangyuan Central Hospital of Sichuan Province, Guangyuan, China; ^4^ Power China Chengdu Engineering Corporation Limited, Chengdu, China; ^5^ Department of Pharmacy, Guanghan People's Hospital, Guanghan, China

**Keywords:** *Brucea javanica*, metabolites, traditional pharmacological effects, modern pharmacological effects, clinical application

## Abstract

This review examines advances in the metabolites, pharmacological research, and therapeutic applications of the medicinal fruit of *Brucea javanica* (L.) Merr. *Brucea javanica* (BJ) is derived from the fruit of the *Brucea javanica* (L.) Merr. There are nearly 200 metabolites present in BJ, and due to the diversity of its metabolites, BJ has a wide range of pharmacological effects. The traditional pharmacological effects of BJ include anti-dysentery, anti-malaria, etc. The research investigating the contemporary pharmacological impacts of BJ mainly focuses on its anti-tumor properties. In the article, the strong monomeric metabolites among these pharmacological effects were preliminarily screened. Regarding the pharmacological mechanism of action, current research has initially explored BJ’s pharmacological agent and molecular signaling pathways. However, a comprehensive system has yet to be established. BJ preparations have been utilized in clinical settings and have demonstrated effectiveness. Nevertheless, clinical research is primarily limited to observational studies, and there is a need for higher-quality research evidence to support its clinical application. There are still many difficulties and obstacles in studying BJ. However, it is indisputable that BJ is a botanical drugs with significant potential for application, and it is expected to have broader global usage.

## Graphical Abstract

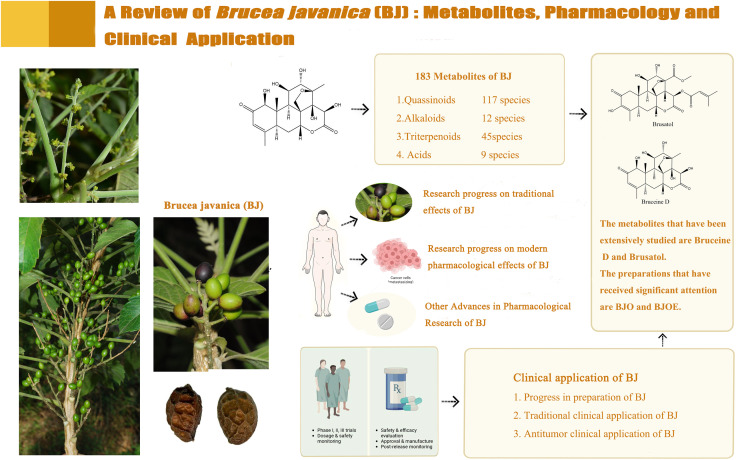

## 1 Introduction

Traditional Chinese Medicine (TCM) has been essential to Chinese healthcare. Chinese botanical drugs (CBD) that incorporate natural compounds are also employed in contemporary medicine to treat several ailments. CBD mainly comprises plant, animal, and mineral medicines, of which plant medicine accounts for the majority. CBD has been widely used in China, some Asian countries, and Europe (Lee et al., 2018; Li et al., 2019a). *Brucea javanica* (L.) Merr. is a well-known CBD belonging to the Simaroubaceae family, an evergreen shrub widely distributed in southeast Asia and northern Australia (Liu et al., 2012a; Zhang et al., 2022), validated by the Plant List database (https://mpns.science.kew.org/mpns-portal/). *Brucea javanica* (BJ) is the fruit of the *Brucea javanica* (L.) Merr., also known as *Yadanzi*, uses ripe dried fruit as medicine. The ripe fruit is black or brown, has a cold nature, and tastes bitter (Chen et al., 2013b; Zhang et al., 2021).

The conventional utilization of BJ in Southeast Asian countries, such as Indonesia and Cambodia, includes its use as an anti-malarial, anti-trypanosomal, and hypoglycemic drug (Murnigsih et al., 2005; NoorShahida et al., 2009; Bawm et al., 2010). In Australia, it is used as an analgesic (Yeshi et al., 2022), while in China, it is used to treat dysentery, malaria, corn, and skin warts (Kitagawa et al., 1994; Yin and Du, 2000; Huang et al., 2017a). The traditional use of BJ has been confirmed through observational studies in numerous research studies (Qiang, 1999; Ge, 2001; Caiyong et al., 2019). There are few studies on the mechanism of action of BJ in traditional applications, and there is a lack of comprehensive literature reviews. With the development of analytical technology, more than 100 metabolites have been isolated from BJ, including quassinoids, triterpenoids, alkaloids, etc. (Kim et al., 2004a; Chen et al., 2011; Chen et al., 2013a; He et al., 2021). In recent decades, extensive studies have demonstrated that the metabolites of BJ possess a diverse range of pharmacological properties, such as anti-cancer, anti-inflammatory, and anti-viral activities (Yang et al., 2013; Ryu et al., 2017; Xie et al., 2021). Most of the research on the metabolites of BJ focuses on quassinoids, which have demonstrated anti-tumor activity in modern research (Cheng et al., 2017; Lee et al., 2020). It is worth noting that alkaloids and acidic metabolites exhibited cytotoxic effects in the study (Toyota et al., 1990; Samat et al., 2017), whereas terpenoids demonstrated anti-inflammatory activity (Hu et al., 2022). This suggests that future research on the metabolites of BJ should not be biased only towards quasinoids. Modern research on the pharmacological mechanisms of action primarily focuses on the anti-tumor effects. Brusatol and Bruceine D are metabolites that have been extensively researched and exert anti-tumor effects through multiple signaling pathways (Xiao et al., 2014; Ye et al., 2018). Notably, multiple metabolites in BJ demonstrate anti-tumor effects through various signaling pathways (Mata-Greenwood et al., 2002; Lu et al., 2021). This indicates that we should not only focus on the mechanisms of well-known metabolites such as Brusatol and Bruceine D. The clinical research on the traditional pharmacological effects of BJ mainly focuses on treating dysentery, skin warts, and corns. The contemporary scientific investigation and practical implementation of BJ primarily focus on its potential as an anti-neoplastic agent. Multiple clinical studies have demonstrated that the co-administration of BJ with other pharmacological agents in individuals with cancer can enhance therapeutic efficacy, improve patients’ quality of life, and mitigate adverse reactions (Shan et al., 2011; Liu et al., 2013; Lu et al., 2013). However, there is a lack of comprehensive literature summarizing clinical research in both traditional and modern applications of BJ.

This review will elaborate on the metabolites of BJ, research on its pharmacological effects, and progress in clinical application research. This paper summarizes the status and challenges of BJ research and suggests future research directions. Fifteen metabolites with research potential were initially identified through current research data.

## 2 Materials and methods

### 2.1 Document retrieval

Search the SciFinder, PubMed, and CNKI databases using the following search methods: SciFinder, PubMed: “All Fields: *Brucea javanica* OR *Yadanzi*,” “All Fields: *Brucea javanica* (L.) Merr. AND *Yadanzi*,” CNKI: “Keywords: Metabolites of *Brucea javanica* (L.) Merr. OR Mechanism of action of *Brucea javanica* (L.) Merr. OR Clinical application of *Brucea javanica* (L.) Merr.,” “Keywords: Metabolites of *Brucea javanica* AND Mechanism of action of *Brucea javanica* AND Clinical application of *Brucea javanica*.”

### 2.2 Inclusion and exclusion criteria

Literature inclusion criteria: research papers and reviews related to BJ. Exclusion criteria: repeatedly published documents, documents with missing critical information such as authors, popular science articles, conference abstracts, news reports, etc. The retrieved documents were imported into the literature manager EndNote software. After deduplication, two researchers screened, read, and analyzed the records according to the inclusion and exclusion criteria and eliminated documents that did not meet the requirements.

### 2.3 Search results

A total of 291 journal articles were obtained from the SciFinder and PubMed databases, while an additional 600 journal articles were collected from CNKI. After removing duplicate articles, excluding popular scientific publications, and articles with missing information, a total of 198 articles were included.

## 3 Metabolites of BJ

This chapter analyses BJ’s metabolites and structure, which contains 183 metabolites. The metabolites include quassinoids, alkaloids, triterpenes, and acids. We will elaborate on the classification of metabolites of BJ.

### 3.1 Quassinoids of BJ

**FIGURE 1:**
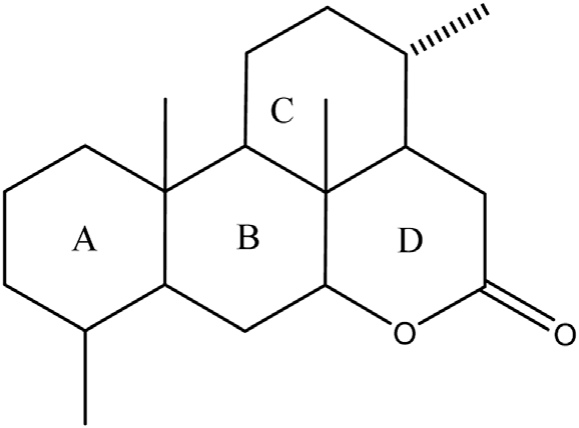
Nuclear parent of quassinoids from BJ.

**FIGURE 2:**
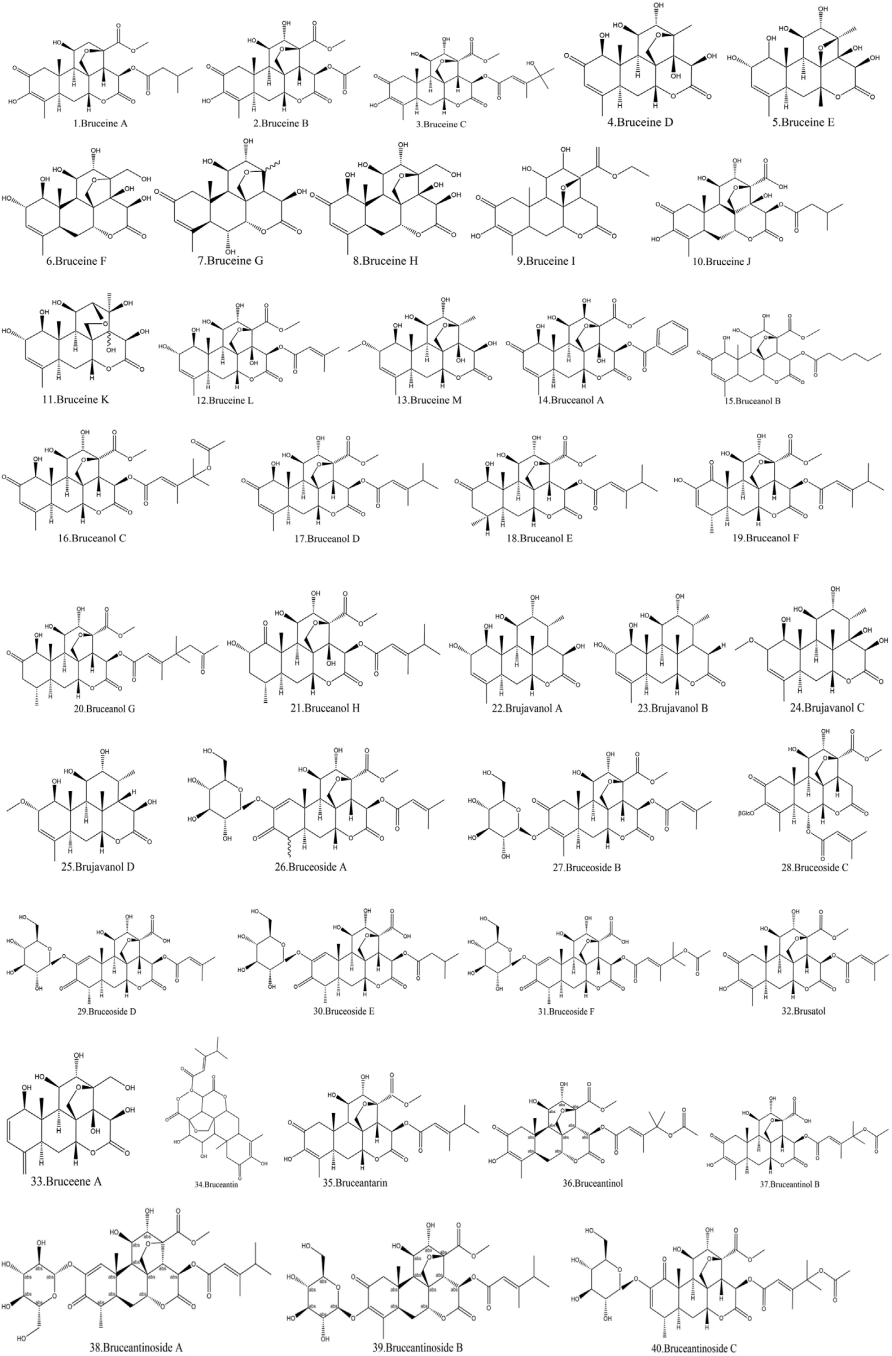
Quassinoids structure diagram from BJ, named with the prefix “Bru.”

**FIGURE 3:**
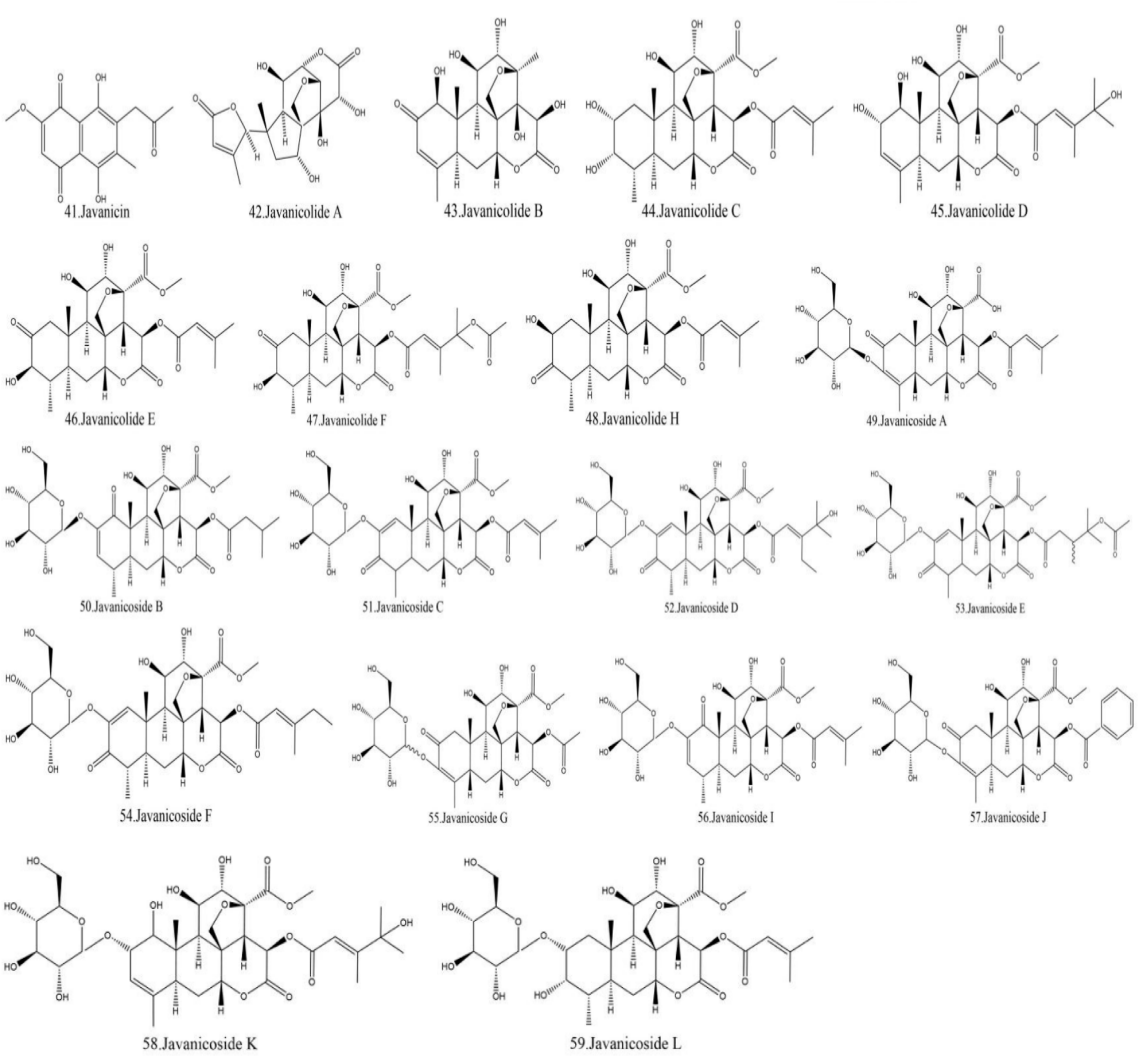
Quassinoids structure diagram from BJ, named with the prefix “Java.”

**FIGURE 4:**
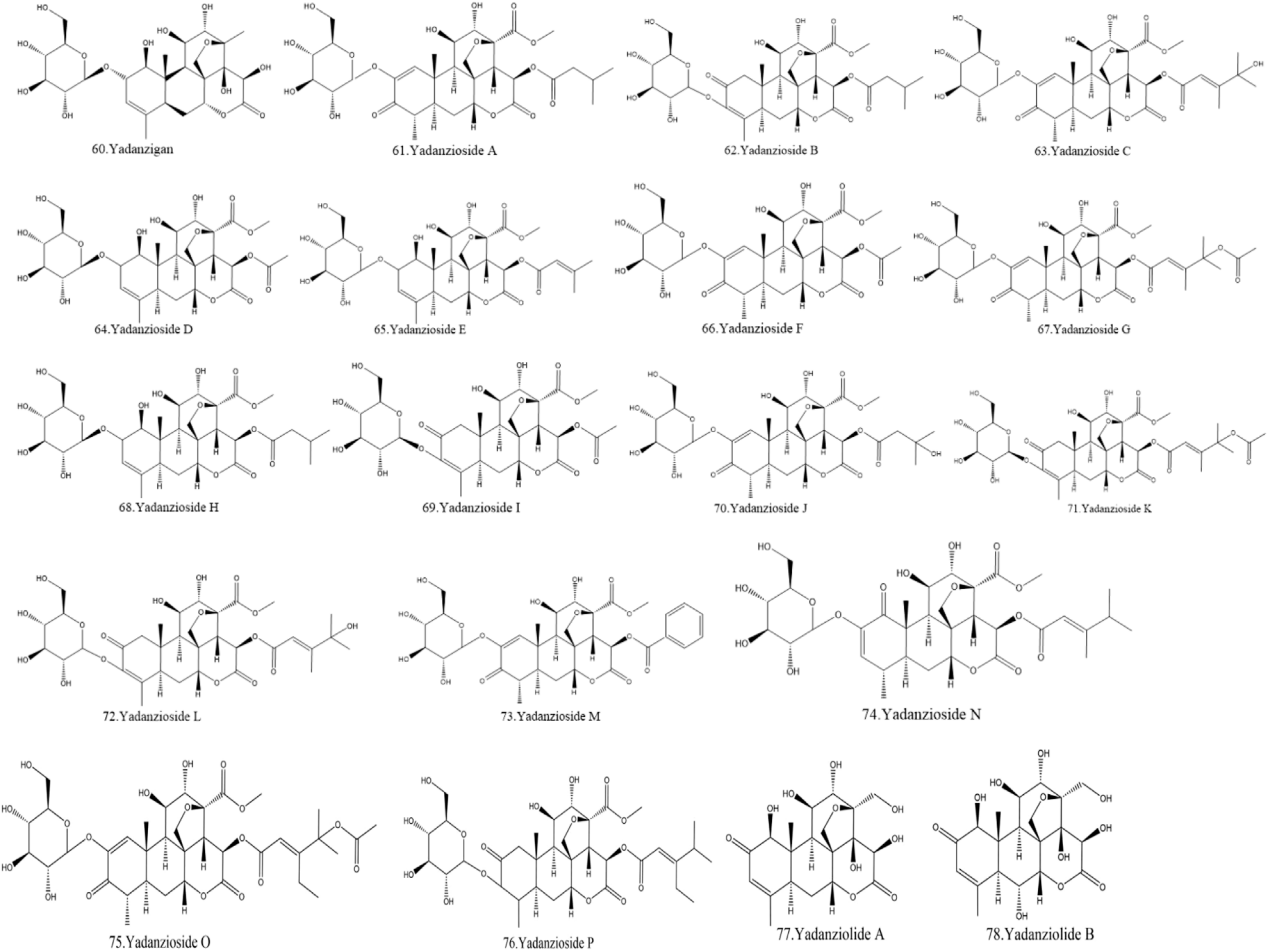
Quassinoids structure diagram from BJ, named with the prefix “Yadanzi.”

**FIGURE 5:**
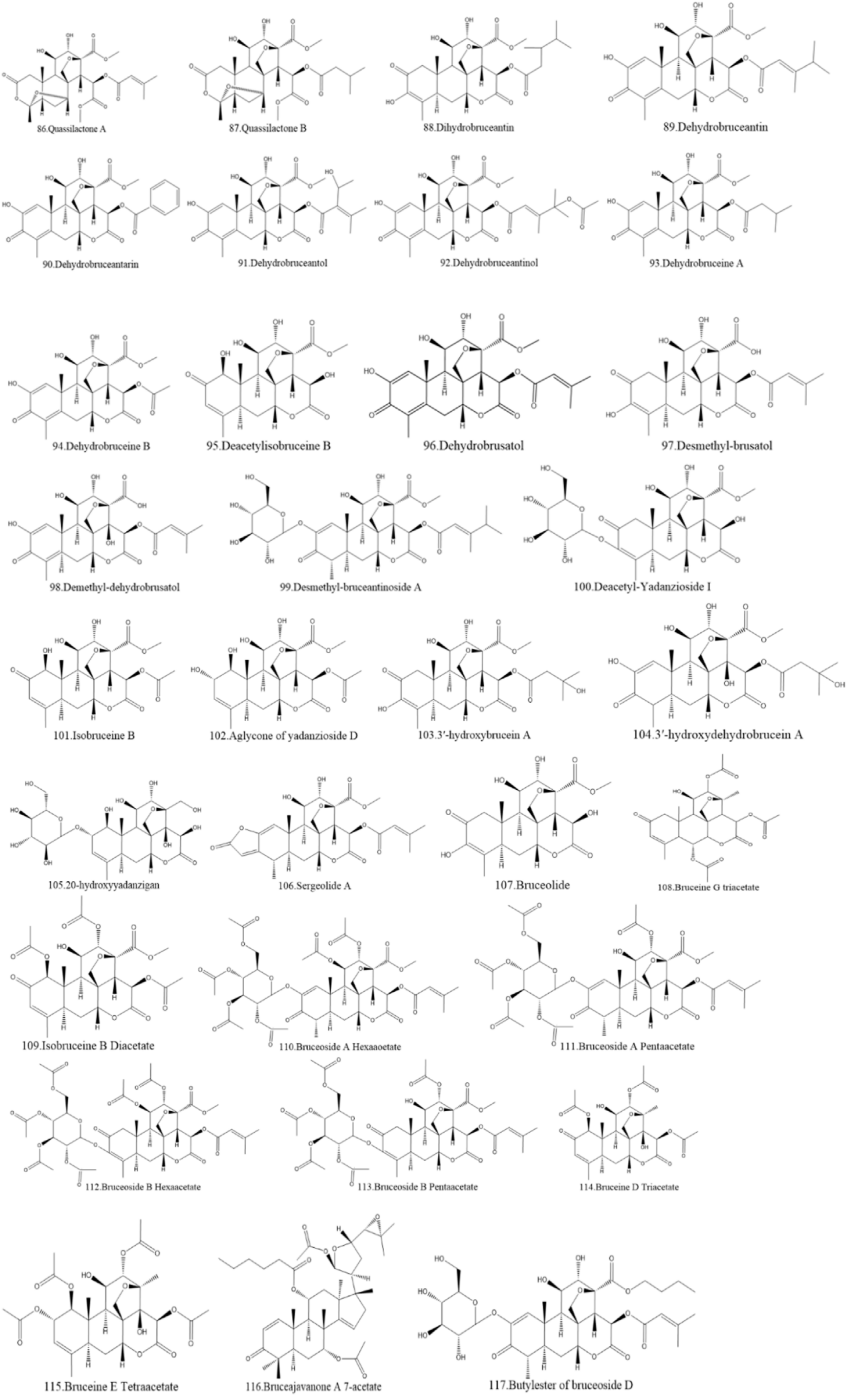
Other quassinoids structure diagram from BJ.

Currently, quassinoids are considered highly significant active metabolites in BJ. Notably, the most notable quassinoids are categorized as C20-quassinoids (Duan et al., 2021). The elemental composition of the core structure includes three hexagonal rings and a lactone ring. Quassinoids exhibit a wide range of pharmacological effects, including traditional applications such as anti-malarial, anti-dysentery, and anti-insect properties (Guo et al., 2005; Bawm et al., 2008; Zhou et al., 2018). In modern research, they also have anti-tumor and anti-muscular atrophy effects (Mata-Greenwood et al., 2002; Baek et al., 2019). In this section, the molecular formulas of quassinoids are presented in tables. There are many quassinoids in BJ. This section classifies and lists the quassinoids based on their naming characteristics. Figure 1 shows the structural diagram of the mother nucleus of quassinoids. Figure 2 displays the structural diagram of quassinoids named with the prefix “Bru”. Figure 3 illustrates the structural diagram of quassinoids named with the prefix “Java”. Figure 4 depicts the structural diagram of quassinoids named with the prefix “Yadanzi”. Lastly, Figure 5 presents the structural diagram of other quassinoids in BJ. Table 1 displays the molecular formula of quassinoids named with the prefix “Bru”. Table 2 illustrates the molecular formula of quassinoids named with the prefix “Java”. Table 3 depicts the molecular formula of quassinoids named with the prefix “Yadanzi”. Lastly, Table 4 presents the molecular formula of other quassinoids in BJ.

**TABLE 1:** Quassinoids from BJ named with the prefix “Bru.”

Name	Formula	Ref.	Name	Formula	Ref.
Bruceine A	C_26_H_34_O_11_	Polonsky et al. (1967)	Bruceine B	C_23_H_28_O_11_	Polonsky et al. (1967)
Bruceine C	C_28_H_36_O_12_	Polonsky et al. (1967)	Bruceine D	C_20_H_26_O_9_	Lee et al. (1979)
Bruceine E	C_20_H_28_O_9_	Lee et al. (1979)	Bruceine F	C_20_H_28_O_10_	Yu and Li (1990)
Bruceine G	C_20_H_26_O_8_	Duncan and Henderson (1968)	Bruceine H	C_20_H_26_O_10_	Zhao et al. (2011a)
Bruceine I	C_22_H_28_O_9_	Yu and Li (1990)	Bruceine J	C_25_H_32_O_11_	Subeki et al. (2007)
Bruceine K	C_20_H_28_O_9_	Zhao et al. (2011b)	Bruceine L	C_26_H_34_O_11_	Zhao et al. (2011b)
Bruceine M	C_21_H_30_O_9_	Su et al. (2013a)	Bruceanol A	C_28_H_30_O_11_	Okano et al. (1985)
Bruceanol B	C_27_H_36_O_11_	Okano et al. (1985)	Bruceanol C	C_30_H_38_O_13_	Fukamiya et al. (1988)
Bruceanol D	C_28_H_36_O_11_	Imamura et al. (1993)	Bruceanol E	C_28_H_38_O_11_	Imamura et al. (1993)
Bruceanol F	C_28_H_36_O_11_	Imamura et al. (1993)	Bruceanol G	C_30_H_40_O_13_	Imamura et al. (1995)
Bruceanol H	C_28_H_38_O_11_	Imamura et al. (1995)	Brujavanol A	C_20_H_30_O_7_	Chumkaew and Srisawat (2016)
Brujavanol B	C_20_H_30_O_6_	Chumkaew and Srisawat (2016)	Brujavanol C	C_21_H_33_O_8_	Chumkaew et al. (2017)
Brujavanol D	C_21_H_32_O_7_	Chumkaew et al. (2017)	Bruceoside A	C_32_H_42_O_16_	Lee et al. (1977)
Bruceoside B	C_32_H_42_O_16_	Lee et al. (1979)	Bruceoside C	C_32_H_42_O_16_	Fukamiya et al. (1992)
Bruceoside D	C_31_H_40_O_16_	Ohnishi et al. (1995)	Bruceoside E	C_31_H_42_O_16_	Ohnishi et al. (1995)
Bruceoside F	C_35_H_46_O_18_	Ohnishi et al. (1995)	Brusatol	C_26_H_32_O_11_	Sim et al. (1968)
Bruceene A	C_20_H_26_O_9_	Ye et al. (2015)	Bruceantin	C_28_H_36_O_11_	Kupchan et al. (1973)
Bruceantarin	C_28_H_30_O_11_	Kupchan et al. (1973)	Bruceantinol	C_30_H_36_O_12_	Kupchan et al. (1975)
Bruceantinol B	C_29_H_35_O_13_	Subeki et al. (2007)	Bruceantinoside A	C_34_H_46_O_16_	Okano et al. (1981)
Bruceantinoside B	C_34_H_46_O_16_	Okano et al. (1981)	Bruceantinoside C	C_36_H_48_O_18_	Fukamiya et al. (1987)

**TABLE 2:** Quassinoids from BJ named with the prefix “Java.”

Name	Formula	Ref.	Name	Formula	Ref.
Javanicin	C_24_H_28_O_12_	Lin et al. (1990)	Javanicolide A	C_19_H_24_O_9_	Kim et al. (2003)
Javanicolide B	C_20_H_26_O_10_	Kim et al. (2003)	Javanicolide C	C_26_H_36_O_11_	Kim et al. (2004b)
Javanicolide D	C_28_H_38_O_12_	Kim et al. (2004b)	Javanicolide E	C_26_H_34_O_11_	Yan et al. (2010)
Javanicolide F	C_30_H_40_O_13_	Yan et al. (2010)	Javanicolide H	C_26_H_34_O_11_	Liu et al. (2012a)
Javanicoside A	C_32_H_42_O_16_	Kim et al. (2003)	Javanicoside B	C_32_H_44_O_16_	Kim et al. (2004b)
Javanicoside C	C_32_H_40_O_16_	Kim et al. (2004b)	Javanicoside D	C_35_H_48_O_17_	Kim et al. (2004b)
Javanicoside E	C_36_H_50_O_18_	Kim et al. (2004b)	Javanicoside F	C_33_H_44_O_16_	Kim et al. (2004b)
Javanicoside G	C_29_H_38_O_16_	He et al. (2021)	Javanicoside I	C_32_H_42_O_16_	Kim et al. (2004a)
Javanicoside J	C_34_H_40_O_16_	Kim et al. (2004a)	Javanicoside K	C_34_H_48_O_17_	Kim et al. (2004a)
Javanicoside L	C_32_H_46_O_16_	Kim et al. (2004a)			

**TABLE 3:** Quassinoids from BJ named with the prefix “Yadanzi.”

Name	Formula	Ref.	Name	Formula	Ref.
Yadanzigan	C_26_H_38_O_4_	Zhang et al. (1983)	Yadanzioside B	C_32_H_44_O_16_	Sakaki et al. (1985)
Yadanzioside A	C_32_H_44_O_16_	Sakaki et al. (1985)	Yadanzioside D	C_23_H_30_O_11_	Sakaki et al. (1985)
Yadanzioside C	C_34_H_46_O_17_	Sakaki et al. (1985)	Yadanzioside F	C_29_H_38_O_16_	Yoshimura et al. (1985)
Yadanzioside E	C_32_H_44_O_16_	Sakaki et al. (1985)	Yadanzioside H	C_32_H_46_O_16_	Sakaki et al. (1985)
Yadanzioside G	C_36_H_48_O_18_	Sakaki et al. (1985)	Yadanzioside J	C_32_H_44_O_17_	Yoshimura et al. (1985)
Yadanzioside I	C_29_H_38_O_16_	Yoshimura et al. (1985)	Yadanzioside L	C_34_H_46_O_17_	Yoshimura et al. (1985)
Yadanzioside K	C_36_H_48_O_18_	Sakaki et al. (1986b)	Yadanzioside N	C_34_H_46_O_16_	Sakaki et al. (1986b)
Yadanzioside M	C_34_H_40_O_16_	Sakaki et al. (1986b)	Yadanzioside P	C_34_H_46_O_16_	Sakaki et al. (1986a)
yadanzioside O	C_31_H_41_O_12_	Sakaki et al. (1986c)	Yadanziolide B	C_20_H_26_O_11_	Yoshimura et al. (1985)
Yadanziolide A	C_20_H_26_O_10_	Yoshimura et al. (1985)	Yadanziolide D	C_19_H_24_O_9_	Yoshimura et al. (1988)
Yadanziolide C	C_20_H_26_O_9_	Yoshimura et al. (1985)	Yadanziolide S	C_20_H_28_O_9_	Su et al. (2002)
Yadanziolide E	C_20_H_26_O_9_	He et al. (2021)	Yadanziolide U	C_26_H_38_O_13_	Chen et al. (2011)
Yadanziolide T	C_20_H_28_O_8_	Chen et al. (2011)	Yadanziolide V	C_19_H_26_O_8_	Chen et al. (2011)

**TABLE 4:** Other quassinoids in BJ.

Name	Formula	Ref.	Name	Formula	Ref.
Quassilactone A	C_26_H_34_O_12_	Su et al. (2020)	Quassilactone B	C_26_H_36_O_12_	Su et al. (2020)
Dihydrobruceantin	C_28_H_38_O_11_	Kupchan et al. (1973)	Dehydrobruceantarin	C_34_H_34_O_14_	Kupchan et al. (1975)
Dehydrobruceantin	C_28_H_40_O_14_	Kupchan et al. (1975)	Dehydrobruceantinol	C_30_H_36_O_13_	Sakaki et al. (1985)
Dehydrobruceantol	C_28_H_34_O_12_	Kupchan et al. (1975)	Dehydrobruceine B	C_26_H_26_O_11_	Kupchan et al. (1975)
Dehydrobruceine A	C_29_H_32_O_11_	Phillipson and Darwish (1981)	Dehydrobrusatol	C_26_H_30_O_11_	Sakaki et al. (1985)
Deacetylisobruceine B	C_21_H_26_O_10_	Okano et al. (1985)	Demethyl-dehydrobrusatol	C_25_H_28_O_11_	He et al. (2021)
Desmethyl-brusatol	C_25_H_30_O_11_	Rahman et al. (1999)	Deacetyl-Yadanzioside I	C_27_H_36_O_15_	He et al. (2021)
Desmethyl-bruceantinoside A	C_33_H_44_O_16_	Rahman et al. (1999)	Aglycone of yadanzioside D	C_23_H_30_O_11_	Kim et al. (2004b)
Isobruceine B	C_21_H_24_O_9_	Okano et al. (1985)	3′-hydroxydehydrobrucein A	C_26_H_35_O_13_	Lahrita et al. (2019)
3′-hydroxybrucein A	C_26_H_35_O_12_	Lahrita et al. (2019)	Sergeolide A	C_28_H_32_O_11_	Yang et al. (2022)
20-hydroxyyadanzigan	C_26_H_38_O_15_	Zhan et al. (2020)	Bruceine G triacetate	C_22_H_28_O_9_	Duncan and Henderson (1968)
Bruceolide	C_21_H_26_O_10_	Polonsky et al. (1967)	Bruceoside A Hexaaoetate	C_44_H_54_O_22_	Lee et al. (1979)
Isobruceine B Diacetate	C_27_H_32_O_13_	Kupchan et al. (1975)	Bruceoside B Hexaacetate	C_44_H_54_O_22_	Lee et al. (1979)
Bruceoside A Pentaacetate	C_42_H_52_O_21_	Lee et al. (1979)	Bruceine D Triacetate	C_26_H_32_O_12_	Lee et al. (1979)
Ruceoside B Pentaacetate	C_42_H_52_O_21_	Lee et al. (1979)	Bruceajavanone A 7-acetate	C_40_H_58_O_9_	Pan et al. (2009)
Bruceine E Tetraacetate	C_28_H_36_O_13_	Lee et al. (1979)	Butylester of bruceoside D	C_35_H_48_O_16_	Rahman et al. (1999)

### 3.2 Alkaloids of BJ

**FIGURE 6:**
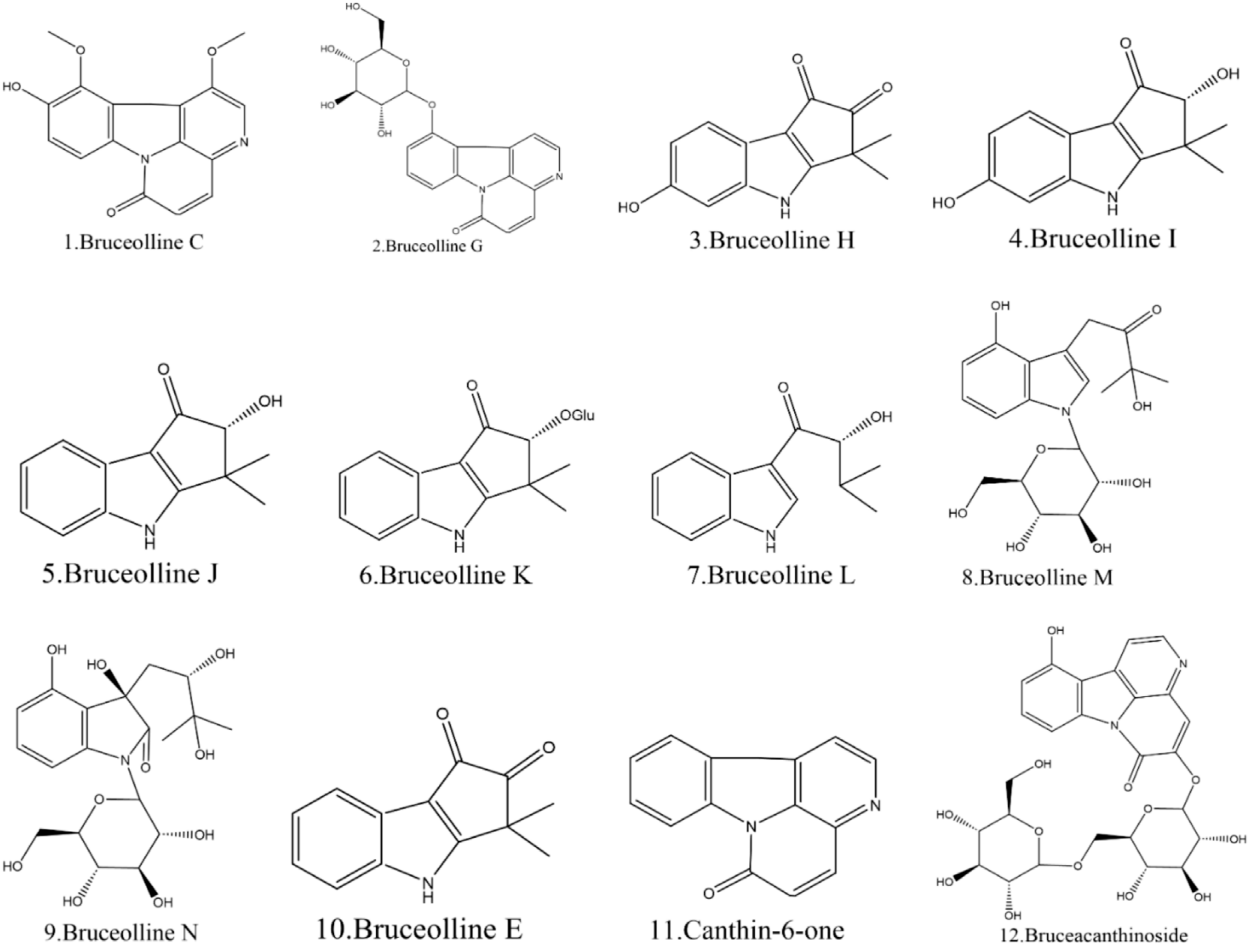
Structural diagram of the alkaloids in BJ.

Currently, 12 alkaloids have been discovered in BJ. Most alkaloids have no documented pharmacological activity. Nevertheless, several alkaloids, such as Canthin-6-one and Bruceolline J, have been discovered to exhibit cytotoxic properties (Samat et al., 2017). Table 5 shows the molecular formula of the alkaloids in BJ. Figure 6 displays the corresponding molecular structure.

**TABLE 5:** Alkaloids of BJ.

Name	Chemical formula	Ref.
Bruceolline C	C_16_H_12_N_2_O_4_	Ouyang et al. (1995)
Bruceolline G	C_20_H_18_N_2_O_7_	Ouyang et al. (1995)
Bruceolline H	C_13_H_11_NO_3_	Chen et al. (2011)
Bruceolline I	C_13_H_13_NO_3_	Chen et al. (2011)
Bruceolline J	C_13_H_13_NO_2_	Chen et al. (2011)
Bruceolline K	C_19_H_23_NO_7_	Chen et al. (2011)
Bruceolline L	C_13_H_15_NO_2_	Chen et al. (2011)
Bruceolline M	C_19_H_25_NO_7_	Chen et al. (2011)
Bruceolline N	C_19_H_27_NO_9_	Chen et al. (2011)
Bruceolline E	C_13_H_11_NO_2_	Jordon et al. (2012)
Canthin-6-one	C_14_H_8_N_2_O	Samat et al. (2017)
Bruceacanthinoside	C_26_H_28_O_12_	Kitagawa et al. (1994)

### 3.3 Triterpenoids of BJ

**FIGURE 7:**
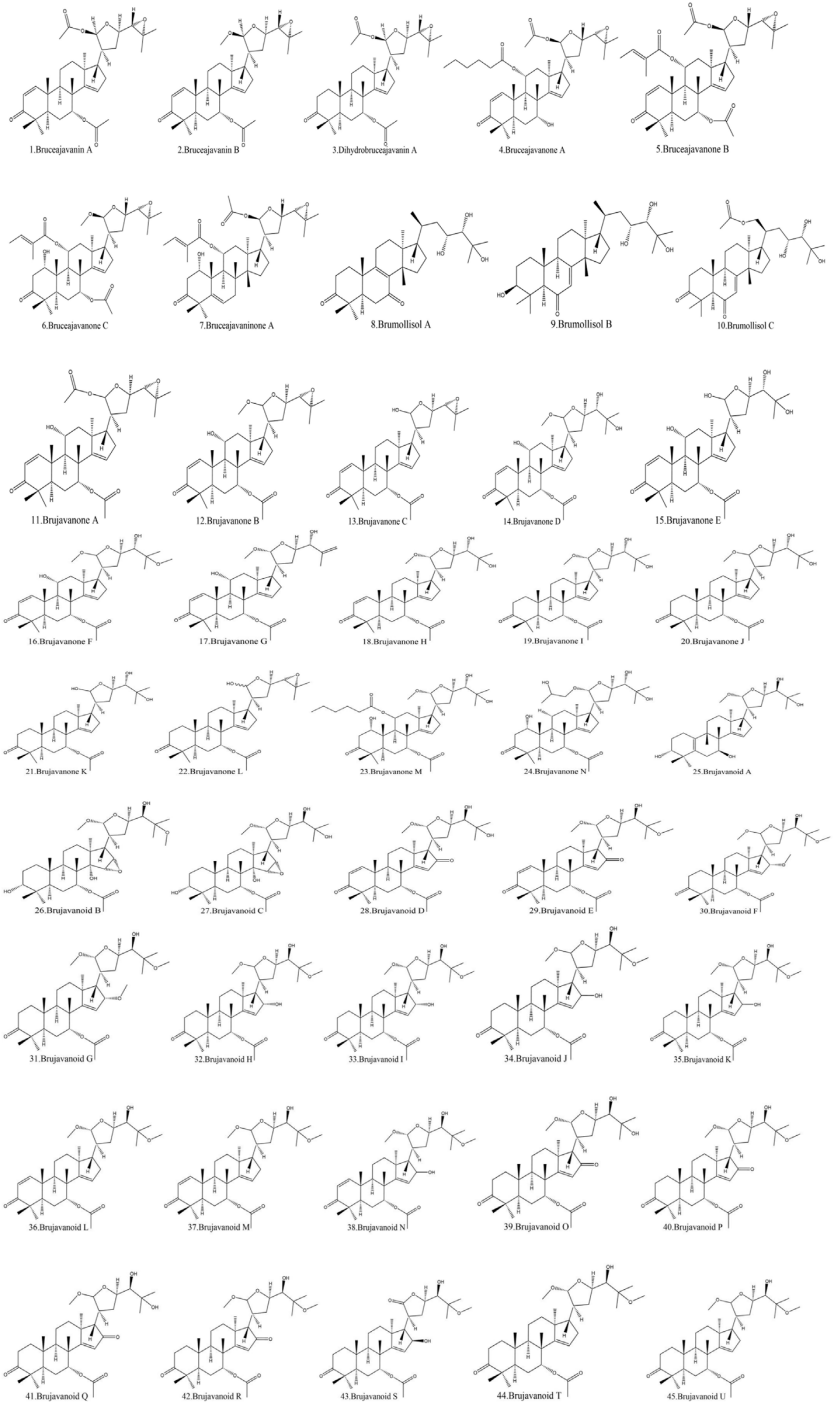
Structural diagram of triterpenoids in BJ.

Another vital metabolite found in BJ is terpenoids. This paper presents a comprehensive list of 45 types of terpenoids documented in the literature. Most of these terpenoids are categorized as pentacyclic triterpenoids, with a lesser fraction falling under tetracyclic triterpenoids. The terpenoids known as brujavanoids A-U have been found to possess anti-inflammatory properties (Hu et al., 2022). Table 6 displays the chemical formulae of terpenoids found in BJ. The molecular structures are seen in Figure 7.

**TABLE 6:** Triterpenoids of BJ.

Name	Formula	Ref.	Name	Formula	Ref.
Bruceajavanin A	C_34_H_48_O_7_	Kitagawa et al. (1994)	Bruceajavanin B	C_33_H_48_O_6_	Kitagawa et al. (1994)
Dihydrobruceajavanin A	C_34_H_50_O_7_	Kitagawa et al. (1994)	Bruceajavanone A	C_36_H_56_O_8_	Pan et al. (2009)
Bruceajavanone B	C_39_H_54_O_9_	Pan et al. (2009)	Bruceajavanone C	C_38_H_56_O_9_	Pan et al. (2009)
Bruceajavaninone A	C_37_H_54_O_8_	Pan et al. (2009)	Brumollisols A	C_30_H_48_O_5_	Chen et al. (2013a)
Brumollisols B	C_30_H_50_O_5_	Chen et al. (2013a)	Brumollisols C	C_32_H_52_O_6_	Chen et al. (2013a)
Brujavanone A	C_34_H_48_O_8_	Dong et al. (2013)	Brujavanone B	C_33_H_48_O_7_	Dong et al. (2013)
Brujavanone C	C_32_H_46_O_6_	Dong et al. (2013)	Brujavanone D	C_33_H_50_O_8_	Dong et al. (2013)
Brujavanone E	C_32_H_48_O_8_	Dong et al. (2013)	Brujavanone F	C_34_H_52_O_8_	Dong et al. (2013)
Brujavanone G	C_33_H_48_O_7_	Dong et al. (2013)	Brujavanone H	C_33_H_50_O_7_	Dong et al. (2013)
Brujavanone I	C_33_H_52_O_7_	Dong et al. (2013)	Brujavanone J	C_33_H_52_O_7_	Dong et al. (2013)
Brujavanone K	C_32_H_50_O_7_	Dong et al. (2013)	Brujavanone L	C_32_H_48_O_6_	Dong et al. (2013)
Brujavanone M	C_39_H_62_O_10_	Dong et al. (2013)	Brujavanone N	C_34_H_54_O_8_	Dong et al. (2013)
Brujavanoid A	C_31_H_50_O_6_	Hu et al. (2022)	Brujavanoid B	C_34_H_54_O_9_	Hu et al. (2022)
Brujavanoid C	C_33_H_52_O_9_	Hu et al. (2022)	Brujavanoid D	C_33_H_48_O_8_	Hu et al. (2022)
Brujavanoid E	C_34_H_50_O_8_	Hu et al. (2022)	Brujavanoid F	C_35_H_56_O_8_	Hu et al. (2022)
Brujavanoid G	C_35_H_56_O_8_	Hu et al. (2022)	Brujavanoid H	C_34_H_54_O_8_	Hu et al. (2022)
Brujavanoid I	C_34_H_54_O_8_	Hu et al. (2022)	Brujavanoid J	C_34_H_54_O_8_	Hu et al. (2022)
Brujavanoid K	C_34_H_54_O_8_	Hu et al. (2022)	Brujavanoid L	C_34_H_54_O_8_	Hu et al. (2022)
Brujavanoid M	C_34_H_52_O_7_	Hu et al. (2022)	Brujavanoid N	C_34_H_52_O_8_	Hu et al. (2022)
Brujavanoid O	C_33_H_50_O_8_	Hu et al. (2022)	Brujavanoid P	C_34_H_52_O_8_	Hu et al. (2022)
Brujavanoid Q	C_33_H_50_O_8_	Hu et al. (2022)	Brujavanoid R	C_34_H_52_O_8_	Hu et al. (2022)
Brujavanoid S	C_33_H_50_O_8_	Hu et al. (2022)	Brujavanoid T	C_34_H_54_O_7_	Hu et al. (2022)
Brujavanoid U	C_34_H_54_O_7_	Hu et al. (2022)			

### 3.4 Acids from BJ

**FIGURE 8:**
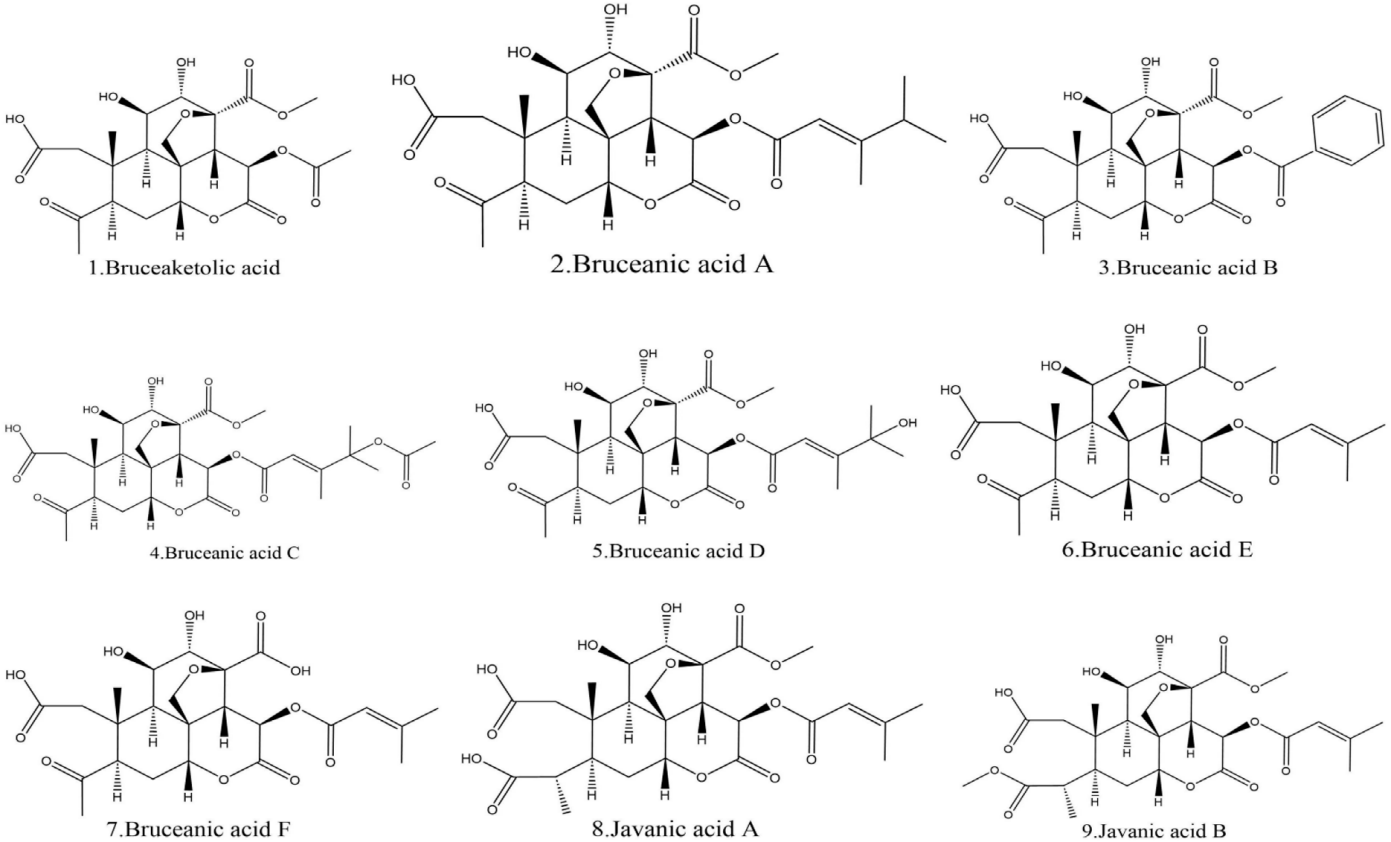
Structural diagram of acids in BJ.

Additionally, this part also lists nine acids found in BJ. Among these acids, Bruceanic acid D (Toyota et al., 1990) exhibited significant cytotoxicity in leukemia. Table 7 provides the molecular structures of the types of acids in BJ. Figure 8 displays the molecular structure.

**TABLE 7:** Acids of BJ.

Name	Chemical formula	Ref.
Bruceaketolic acid	C_22_H_26_O_11_	Lin et al. (1990)
Bruceanic acid A	C_27_H_36_O_12_	Toyota et al. (1990)
Bruceanic acid B	C_27_H_30_O_12_	Toyota et al. (1990)
Bruceanic acid C	C_29_H_38_O_14_	Toyota et al. (1990)
Bruceanic acid D	C_27_H_36_O_13_	Toyota et al. (1990)
Bruceanic acid E	C_25_H_32_O_12_	Liu et al. (2012a)
Bruceanic acid F	C_24_H_30_O_12_	Liu et al. (2012a)
Javanic acid A	C_26_H_34_O_13_	Liu et al. (2012a)
Javanic acid B	C_27_H_36_O_13_	Liu et al. (2012a)

## 4 Advances in BJ’s pharmacology

The metabolites of BJ are diverse and exhibit a wide range of pharmacological effects. This review summarizes the research progress on the pharmacological mechanism of BJ in traditional usage and its molecular mechanism of action in modern applications. We initially addressed the research advancements regarding the traditional pharmacological properties of BJ, including its anti-dysentery, anti-malaria, insecticidal, skin wart, and corn-fighting actions. Then, we discussed the latest advancements in research on the contemporary pharmacological effects of BJ, specifically focusing on its anti-tumor properties. The objective is to provide a research foundation for its future clinical utilization.

### 4.1 Research progress on traditional effects of BJ

**FIGURE 9:**
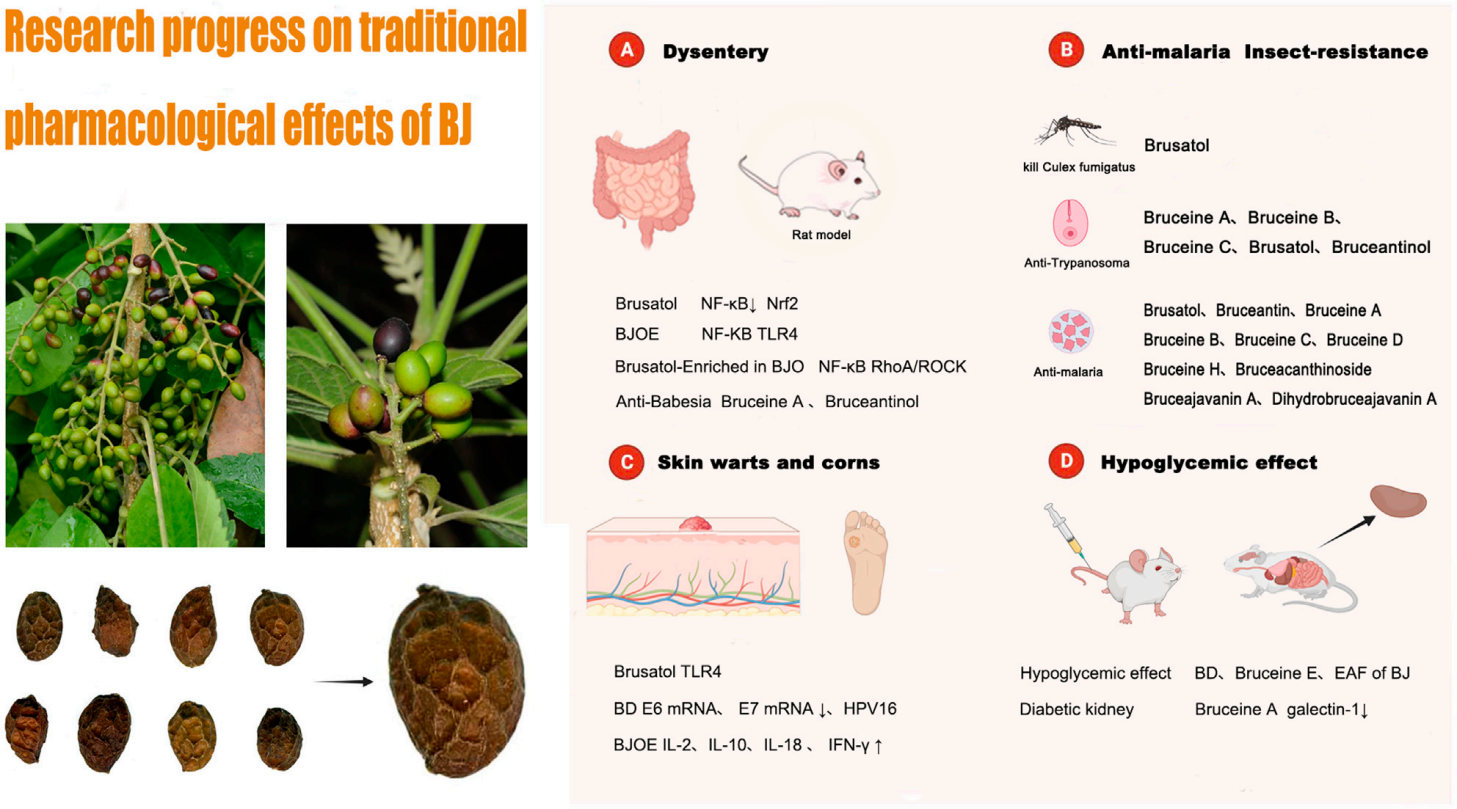
The pharmacological mechanism of BJ’s traditional application.

In this section, we will describe the research progress on the traditional pharmacological effects of BJ. These effects include its anti-dysentery, anti-malarial, and insecticidal, as well as its use in the treatment of skin warts and corns. We have initially screened out compounds with pharmacologically active effects in traditional applications and their corresponding pharmacological action mechanisms, hoping to provide valuable information for future research. Research progress on the pharmacological mechanism of BJ’s traditional application is depicted in Figure 9. It should be noted that the traditional pharmacological mechanism of BJ, whether it is used for anti-inflammatory diseases, reducing blood sugar levels, or addressing skin conditions such as warts and corns, still requires further understanding. Current research is primarily limited to *in vitro* and animal studies, and there is a lack of high-quality clinical research evidence in this area. Therefore, it is necessary to further study the mechanisms behind the traditional pharmacological effects of BJ and conduct scientific and rigorous clinical research. This is the cornerstone for further promoting BJ on a global scale.

#### 4.1.1 Anti-dysentery effect of BJ

BJ, as a remedy for diarrhea, is used in traditional Southeast Asian medicine. In modern medicine, “dysentery” encompasses ulcerative colitis and bacillary dysentery (Yu et al., 2023). The primary causes of dysentery are bacterial and amoebic infections (Siciliano et al., 2021), which primarily result in gastroenteritis and ulcerative gastroenteritis. The potential efficacy of BJ in treating dysentery may stem from its antibacterial and anti-inflammatory characteristics. In this section, we will present the metabolites of BJ used for treating dysentery and provide an overview of the advancements in pharmacological research in this field.

Numerous metabolites present in BJ have exhibited significant anti-inflammatory properties in various research studies. Studies have been undertaken to investigate the underlying mechanism responsible for the anti-inflammatory activities exhibited by these metabolites. Brusatol (Zhou et al., 2018) has been shown to reduce inflammation in RAW264.7 cells stimulated by lipopolysaccharide and in rats with colitis induced by 2,4,6-trinitrobenzenesulfonic acid. This effect may be ascribed to the decrease in levels of inflammatory cytokines, which is associated with the suppression of the NF-κBNF-ΚB signaling pathway and the modulation of Nrf2-mediated oxidative stress. Brusatol-enriched in Brucea javanica oil (BJO) (Zheng et al., 2021) improved dextran sodium sulfate-induced enteritis, which may be related to the inhibition of NF-ΚB and ras homolog gene families, member A/rho-associated kinase (RhoA/ROCK) signaling pathways. The anti-inflammatory benefits of Brucea javanica oil emulsion (BJOE) on ulcerative colitis in mice are significant. This may be attributed to its ability to block the NF-ΚB signal transduction pathway and reduce the expression of inflammatory mediators (Huang et al., 2017a; Li et al., 2018). Huang et al. (2019) further revealed that BJOE was superior to sulfasalazine and azathioprine in the treatment of colitis induced by 2,4,6-trinitrobenzene sulfonic acid in rats. The observed phenomenon could be linked to the suppression of the toll-like receptor 4 (TLR4)-associated NF-κB signaling pathway, which results in a subsequent decrease in inflammatory mediators.

Furthermore, BJ exhibits pharmacological effects against amoeba. The amoeba infiltrates the human intestinal tract, resulting in the onset of a condition known as amoebic dysentery. A study conducted in Indonesia revealed that bruceine A and bruceantinol have demonstrated significant anti-amoeba activity *in vitro*, as evidenced by their IC50 values of 0.01 and 0.02 μmol/L, respectively (Subeki et al., 2007).

In summary, the monomeric metabolites of BJ, including Brusatol, Bruceine A, and Bruceantinol (Subeki et al., 2007; Zhou et al., 2018), have shown promising properties as anti-dysenteric agents. Among them, Brusatol has initially been explored for its mechanism of action in research on inflammatory diseases. Qian et al. (2011) demonstrated that the aqueous extract of BJ (AE-BJ) exhibits a more potent anti-inflammatory effect compared to the alcohol-extracted metabolite and shows a good dose-effect relationship. Based on the studies, Brusatol and the aqueous extract of BJ may serve as indicators of BJ’s anti-inflammatory effect. Nonetheless, existing research mainly consists of *in vitro* and animal studies, and research on the molecular mechanism of action is still in the early stages of exploration. It is still necessary to further study the mechanism of BJ’s anti-dysentery effect and elucidate its potential mechanisms to lay the foundation for subsequent research in this field.

#### 4.1.2 Antimalarial and insecticidal effects of BJ

BJ has anti-plasmodium activity and is often used in traditional applications for the treatment of malaria. The anti-insect effects of BJ mainly include anti-Plasmodium, anti-Babesia, anti-Trypanosoma, and the ability to kill Culex fumigatus. Among the metabolites of BJ, Brusatol exhibits various functions, including anti-malarial, anti-trypanosome, and mosquito-killing properties against Culex mosquitoes. Among the metabolites investigated, Bruceantin exhibits the most potent antimalarial activity (Guo et al., 2005), consistent with previous findings from the University of London School of Pharmacy (Oneill et al., 1988). Based on current research, it is speculated that Bruceantin may serve as a potential antimalarial metabolite for BJ. Table 8 lists metabolites of BJ with insecticidal properties.

**TABLE 8:** Metabolites in BJ with anti-insect and anti-malarial effects.

Pharmacological effect	Metabolites	Type of study	Quantitative indicators	Ref.
Anti-malaria	Bruceine D	Plasmodium K1	1.41 μmol/L/IC50	Chumkaew and Srisawat (2016)
	Bruceine H	Plasmodium K1	1.06 μmol/L/IC50	Chumkaew et al. (2017)
	Bruceajavanin A	Plasmodium K1	1.10 μmol/L/IC50	Kitagawa et al. (1994)
	Dihydrobruceajavanin A	Plasmodium K1	2.50 μmol/L/IC50	Kitagawa et al. (1994)
	Bruceacanthinoside	Plasmodium K1	25.00 μmol/L/IC50	Kitagawa et al. (1994)
	Bruceine A	P. falciparum	0.02 μmol/L/IC50	Guo et al. (2005)
	Bruceine B	P. falciparum	0.02 μmol/L/IC50	Guo et al. (2005)
	Bruceine C	P. falciparum	0.01 μmol/L/IC50	Guo et al. (2005)
	Brusatol	P. falciparum	0.01 μmol/L/IC50	Guo et al. (2005)
	Bruceantin	P. falciparum	0.002 μmol/L/IC50	Guo et al. (2005)
Anti-Trypanosoma	Bruceine A	Vitro	2.90 μmol/L/IC50	Bawm et al. (2008)
	Bruceine B	Vitro	17.80 μmol/L/IC50	Bawm et al. (2008)
	Bruceine C	Vitro	4.80 μmol/L/IC50	Bawm et al. (2008)
	Brusatol	Vitro	13.60 μmol/L/IC50	Bawm et al. (2008)
	Bruceantinol	Vitro	6.50 nmol/L/IC50	Bawm et al. (2008)
Kill Culex fumigatus	Brusatol	Culex fumigatus	0.654 ± 0.081/ppm/LC90	Dwi Sutiningsih et al. (2019)

#### 4.1.3 Skin warts and corn treatment of BJ

Warts are benign skin growths caused by a deoxyribonucleic acid (DNA) virus known as the human papillomavirus (HPV), which infects the outer layer of the skin and enters the epithelial cells. BJ monomer metabolites and extracts have shown potential effects in treating genital warts in research studies. The mechanisms of action of BD and brusatol for treating genital warts have been demonstrated in in vitro studies. BD and brusatol may be effective metabolites for the future treatment of genital warts. Corns are keratoproliferative lesions that appear as localized cone-shaped growths on the skin of the feet (Yuchen et al., 2017). BJ has been found to possess inhibitory effects on HPV and a corrosive impact on the skin, making it a potential treatment option for skin warts and corns.

Lianna et al. (2014) demonstrated the potential of BJOE for treating condyloma acuminatum. They observed that the injection of BJOE increased the levels of serum interleukin-2, interleukin-10, interleukin-18, and interferon-r. Bruceine D (BD) has shown inhibitory effects on HPV-16 infection in multiple studies (Liuliu et al., 2015; Jingli et al., 2017). BD may hinder the continued growth of virus-infected cells by reducing the expression of E6 and E7 mRNA in cells infected with high-risk HPV16. In addition, Brusatol can regulate the expression of Toll-like receptor 4 (TLR4) and inhibit the proliferation of genital warts keratinocytes, thereby regulating its progression (Weiqi et al., 2023). BJ is also utilized for the treatment of corn. It is crushed and applied topically to the affected area (Xiongzhang and Guang, 2000). However, the specific mechanism of action has not been reported.

#### 4.1.4 Diabetes and diabetic nephropathy application of BJ

In the context of traditional medicinal practices in Malaysia, it has been observed that BJ is used as a therapeutic intervention for managing diabetes. In research investigations, various active compounds found in BJ have been shown to possess pharmacological actions that lower blood glucose levels. BD and Bruceine E (NoorShahida et al., 2009) were found to significantly reduce glucose levels in both streptozotocin-induced diabetic rats and normoglycemic mice. The reduction observed in diabetic rats was approximately 40.07% ± 11.45%, while in normoglycemic mice, it was approximately 48.82% ± 13.34%. Additionally, the blood glucose decreases curves observed for BD and Bruceine E were similar to those of the positive control, glibenclamide. The blood glucose level of rats was shown to decrease upon administration of the ethyl acetate fraction (EAF) of BJ at a dosage of 125 mg/kg (Ablat et al., 2014). The blood glucose level decreased by 39.91% after 30 min, followed by a further decline of 28.89% and 20.29% at 60 min and 90 min, respectively. When exposed to high glucose stress, Bruceine A has been found to inhibit galectin-1 in rat mesangial HBZY-1 cells. This finding implies that Bruceine A has the potential to function as a renoprotective agent in a murine model of diabetic nephropathy (Li et al., 2022).

The monomer metabolites of BJ, BD, Bruceine E, Bruceine A, and the EAF of BJ all exhibit hypoglycemic effects. Among them, the hypoglycemic effects of BD and Bruceine E are stronger than the EAF of BJ. It is speculated that BD and Bruceine E may be potential compounds for future hypoglycemic effects.

### 4.2 Research progress on modern pharmacological effects of BJ

Modern pharmacological research on BJ has found various pharmacological effects, including anti-tumor, anti-tuberculosis, anti-muscle atrophy, and lipolysis. Among them, the anti-tumor properties are of the most significant interest. In this section, we screen out compounds in BJ that have pharmacological activities, such as anti-tumor and anti-muscle atrophy, and at the same time, clarify the research progress of the corresponding pharmacological mechanism of action, laying the foundation for future research.

#### 4.2.1 Lymphocytic leukemia

Individual metabolites of BJ, including Bruceanol D, Brusatol, Bruceantin, and Yadanzioside O, have shown potential for further exploration in lymphocytic leukemia. The primary focus of research on compound preparations predominantly revolves around BJO. *In vitro* P-388 lymphocytic leukemia cells, Bruceoside C (Fukamiya et al., 1992), Yadanzioside G (Fukamiya et al., 1987), Bruceantinoside C (Fukamiya et al., 1987), and Yadanzioside N (Fukamiya et al., 1987) demonstrated potent cytotoxicity with ED50 values of 3.15–7.49 μmol/L. Bruceanic acid D (Toyota et al., 1990), Bruceanol C (Fukamiya et al., 1988), Bruceanol E, Bruceanol F, and Bruceanol D (Imamura et al., 1993) demonstrated significant cytotoxicity with ED50 values of 0.16–1.36 μmol/L. From the above metabolites, Bruceanol D has a higher safety rating. Javanicoside I, J, K, and L (Kim et al., 2004a) and Javanicoside B (Kim et al., 2004b) showed moderate cytotoxic activity against P-388 murine leukemia cells, with IC50 values ranging from 0.68 to 0.77 μmol/L. Through *in vitro* tests on the HL-60 cell line, Liu et al. demonstrated that Brusatol and Bruceine B (Liu et al., 2011) exhibited significant inhibitory activity against HL-60 cells, with IC50 values of 0.06 and 0.27 μmol/L, respectively. Brusatol and Bruceantin (Mata-Greenwood et al., 2002) exerted significant cytotoxic effects on several leukemic cell lines, showing IC50 values in the range of 0.01–0.19, 0.003–0.096 μmol/L, respectively. The results showed that Brusatol and Bruceantin (Mata-Greenwood et al., 2002) had the strongest cytotoxicity against the Daudi cell line, with IC50 values of 0.01 ± 0.001 ng/mL and 0.003 ± 0.0002 μmol/L. In the compound extract study, BJO exhibited cytotoxicity towards Hl-60 and U937 cells, with IC50 values of 312.7 and 265.4 μg/mL, respectively (Zhang et al., 2011).

*In vivo* studies of mouse P388 lymphocytic leukemia, Yadanzioside A, B, C, D, E, and G showed antileukemic activity at a dose level of 10 mg/kg, with increased lifespan (ILS) values in experimental mice of 2.0%–9.2% (Sakaki et al., 1985). Yadanzioside P (Sakaki et al., 1986a) exhibited antileukemic activity at doses of 5 and 10 mg/kg/day, increasing ILS values of 15.5% and 28.9% in mice, respectively. Yadanzioside O exhibited antileukemic activity at 2 mg/kg/day doses and 4 mg/kg/day, resulting in a 37.1% and 47.2% increase in mouse ILS values, respectively (Sakaki et al., 1986b). Yadanzioside O, the monomeric metabolite of BJ, showed the best survival advantage in animal studies.

In the investigation of mechanisms of action, Brusatol and Bruceantin (Mata-Greenwood et al., 2002) inhibited cell proliferation by primarily blocking G0/G1 phase cells. They may trigger cell cycle arrest by downregulating the expression of the cellular myeloma viral oncogene (c-MYC), thereby exerting anti-leukemia effects. Additionally, Brusatol can downregulate the expression of c-MYC in normal lymphocytes. The mechanism of action of BJO and BJOE in leukemia has been partially confirmed in research. This includes inhibiting the activation of the phosphoinositide 3-kinase/protein kinase B (PI3K/Akt) signaling pathway and upregulating its downstream targets, such as p53 and forkhead box genes, to initiate apoptosis., In addition, apoptosis was induced through the mitochondrial and death receptor apoptosis pathways by downregulating the expression of c-FLIP(L/S), myeloid cell leukemia-1, Bcl-2 (B-cell lymphoma-2), and XIAP (Zhang et al., 2011; Zhang et al., 2021).

#### 4.2.2 Lung cancer

Several metabolites found in BJ exhibit notable cytotoxic properties against lung cancer. The substances Brusatol, Bruceloside C, and BD exhibit promise for further investigation in the context of lung cancer. Additionally, the composite extract AE-BJ demonstrates potential for further exploration in the field of lung cancer research.

Bruceanol E, D, F (Imamura et al., 1993), Bruceanol C (Fukamiya et al., 1988), and Bruceoside C (Fukamiya et al., 1992) exhibited cytotoxicity against A549 lung carcinoma, with ED50 values of 0.65–6.82 μmol/L. Bruceloside C has a minimum ED50 of 0.65 μmol/L, suggesting that it is a safer option compared to other monomers. Bruceosides D, E, and F (Ohnishi et al., 1995) showed selective cytotoxicity in non-small cell lung cancer cell lines, with log GI50 values ranging from −4.14 to −5.66. Quassilactones A and B (Su et al., 2020) exhibited cytotoxic activity against A549 cells with IC50 values of 66.43 ± 0.15 and 75.67 ± 0.10 μmol/L, respectively. BD (Tan et al., 2019) inhibited the viability of H460 and A549 cells with IC50 values of 0.5 and 0.6 μmol/L, respectively. Brusatol (Liu et al., 2011), Bruceine B (Liu et al., 2011), 24-epipiscidinol A (Chen et al., 2013a), and Yadanziolide B (Chen et al., 2011) demonstrated cytotoxicity against human A549 cells. Brusatol exhibited the highest level of cytotoxicity among these metabolites, with IC50 values of less than 0.0064 μmol/L. Studies on the preparation of BJ, Ethanol Extract of BJ, Petroleum Ether Extract of BJ and Ethyl acetate Extract of BJ (BJE), and n-butyl alcohol Extract of BJ (Su et al., 2013b) showed significant cytotoxicity against A549 cell lines, with an IC50 value ranging from 0.02 to 17.47 μg/mL. The warmed water extract of BJ (Lau et al., 2005) induced cell death in non-small-cell lung carcinoma (NSCLC) A549 with an IC50 of 50 ug/mL. The crude extract and its metabolites (Bruceacanthinones A, B et al.) (Makong et al., 2019) exhibited cytotoxicity, with IC50 values ranging from 50.0 ± 5.2 to 80.5 ± 1.8 μg/mL, with a single metabolite showing less cytotoxicity than the crude extract. Aqueous BJ extract (Kim et al., 2016) inhibited the growth of H1975 cells with an IC50 of 2,000 μg/mL while being the specific in NSCLC cells bearing L858R/T790M epidermal growth factor receptor (EGFR).

The investigation into the metabolites of BJ in lung cancer demonstrated that brusatol and dehydrobruceine B (DHB) effectively increased the concentration of reactive oxygen species (ROS) by activating the NRF2 pathway (Zhao et al., 2016; Xie et al., 2021). Furthermore, it activates the pro-apoptotic signal through the mitochondrial intrinsic pathway, resulting in the initiation apoptosis. BD is another monomer metabolite that can induce lung cancer cells through the same mechanism (Xie et al., 2019). BD also promotes apoptosis in lung cancer cells via the JNK pathway (Yang et al., 2013). In addition, it was observed that the AE-BJ [101] exhibited inhibitory effects on the proliferation of H1975 spheroid cells. AE-BJ was also found to reduce the proliferation of stem cells and enhance their apoptotic characteristics (Kim et al., 2021). It has been demonstrated that the metabolite exhibited inhibitory effects on the growth of implanted xenograft H1975 tumors, while simultaneously preserving the overall wellbeing of the animals. The observed inhibition may have something to do with apoptosis and stopping the growth of tumours, specifically in (NSCLC) with somatic epidermal EGFR mutations (Kim et al., 2016). Another preparation of BJ, BJO, induced apoptosis in A549 and H446 lung cancer cells, possibly molecularly mediated by the mitochondrial/caspase pathway by increasing ROS production (Wang et al., 2016a).

#### 4.2.3 Cancer of the digestive system

##### 4.2.3.1 Hepatocellular carcinoma (HCC)

Studies on liver cancer have shown that Brusatol, BD, and Bruceine B exhibit potent cytotoxic effects. Among them, the mechanisms of action of Brusatol and BD have also been preliminarily studied and are valuable for further research. The preparations BJO and AE-BJ also have the potential for further research.

The metabolites of BJ in liver cancer cells have primarily been investigated in Bel-7404, HEPG2, and SMMC 7721 cell lines. Among the metabolites tested, Brusatol exhibited the most potent cytotoxic effect in the SMMC 7721 cell line. Yadanziolide T and Yadanziolide B (Chen et al., 2011) exhibited cytotoxicity against Bel-7402 cells, with IC50 values of 3.5–4.5 μmol/L. Bruceantinol (Su et al., 2013a) had significant growth-inhibiting activity on Bel-7404 with an IC50 < 10 μmol/L. Javanicolide H, Bruceine B, Bruceine E, Bruceine H, Dehydrobrusatol, and Javanicolide E (Liu et al., 2012a) exhibited cytotoxicity against HEPG2 with IC50 values between 0.81 and 3.3 μmol/L. BD demonstrated notable cytotoxicity in HEPG2 cells, with an IC50 value of 1.2 μmol/L (Liu et al., 2012a). Bruceine B (Liu et al., 2011) had significant inhibitory activity against SMMC 7721 hepatocellular carcinoma cells with an IC50 of 0.15 μmol/L. Brusatol demonstrated inhibitory effects on cell viability in SMMC-7721, Huh7, and Hep3B liver cancer cells, with IC50 values of less than 0.064, 0.34, and 0.69 μmol/L, respectively (Liu et al., 2011; Ye et al., 2018). Additionally, it exhibited inhibitory effects on tumor invasion and migration. The IC50 value of BJ’s aqueous extract (Chen et al., 2016) on Hep3B cells was found to be 4 mg/mL. It is worth noting that this extract not only promoted apoptosis in hepatoma cells but also inhibited the growth of derived stem cell-like cells. BJ water extract (Lau et al., 2005) was found to induce cell death in Hep3b cells with an IC50 of approximately 50 ug/mL.

Studies have found that BD and Brusatol exert anti-liver cancer effects through multiple signaling pathways. BD significantly inhibited tumor growth in mice with Hep3B, possibly by regulating the expression of miR-95 (Xiao et al., 2014). Another study found that BD affects the breakdown of proteins and the removal of β-catenin. It may also work as a Wnt/Notch crosstalk inhibitor, which means it works better with sorafenib to fight tumors (Cheng et al., 2017). Brusatol has exhibited notable effects in inhibiting proliferation, invasion, and metastasis in a murine model of liver transplantation tumor in humans. These effects are thought to happen through the PI3K/Akt/mTOR pathway and by messing with STAT3 (Ye et al., 2018; Lee et al., 2020).

In the preparation research of BJ, AE-BJ induced death of hepatocarcinoma cells may be related to the mitochondria-dependent pathway associated with caspase 3 activation (Lau et al., 2005). BJO (Shi et al., 2015) inhibited tumor growth in H22 mice, which could be linked to the degradation of energy metabolism, tumor proliferation, and immunomodulatory activity mediated by Akt. Another BJO preparation, Brusatol-BJO (Wang et al., 2021) significantly increased the survival time of H22 ascites tumor-bearing mice, which could be linked to miRNA-29b and the mitochondria-associated pathway.

##### 4.2.3.2 Other cancers of the digestive system

In gastrointestinal cancer research, several metabolites have demonstrated noteworthy cytotoxic effects and show promise for further investigation. Specifically, Bruceanol G has shown potential in oral cancer, while Brusatol has demonstrated promise in laryngeal cancer. Additionally, BD and Javanicolide H have displayed significant cytotoxic effects in gastric cancer. In pancreatic cancer, both Bruceine A and Brusatol have demonstrated potential therapeutic effects. BD and Brusatol have been investigated for their mechanisms of action in colorectal cancer, while the primary focus of research on preparations revolves around BJOE.

BJ phytochemicals have shown cytotoxic effects on oral cancer, laryngeal cancer, gastric cancer, and intestinal cancer cell lines *in vitro* studies, which we elaborated on in different tumors. Brujavanol A and B (Chumkaew and Srisawat, 2016) showed significant cytotoxicity to human oral cancer cells, with IC50 values of 3.40 and 6.45 μmol/L, respectively. Brujavanol E (Chumkaew et al., 2019) and Bruceanol G (Imamura et al., 1995) exhibited cytotoxic activity against human oral squamous cell carcinoma with IC50 values of 5.54 and 0.55 μmol/L, respectively. BJ extract (Majid et al., 2014) had anti-proliferative activity on KB and ORL-48 cells with IC50 of 24.37 ± 1.75 and 6.67 ± 1.15 ug/mL, respectively. Bruceanol G demonstrated excellent cytotoxicity in oral cancer, while BJ extract demonstrated superior anti-proliferative activity.

*In vitro* studies of throat cancer, Brusatol (Zhou et al., 2021) demonstrated substantial cytotoxicity to the human laryngeal cancer cell line Hep-2 in the investigation of throat cancer, with an IC50 of 0.53 μmol/L. The IC50 of AE-BJ (Lau et al., 2005) on esophageal squamous cell carcinoma SLMT-1 was around 50 ug/mL.

In gastric cancer cells, Yadanziolides T, BD, yadanziolide B (Chen et al., 2011), Javanicolide H, Bruceine B, D, E, H, Dehydrobrusatol, Javanicolide E (Liu et al., 2012a), and 24-epipiscidinol A (Chen et al., 2013a) exhibited cytotoxicity against BGC-823 cells with IC50 values of 0.52–4.85 μmol/L. Among the monomers, Javanicolide H showed the strongest effect on the BGC-823 gastric cancer cell line, with an IC50 of 0.52 μmol/L.

*In vitro* studies of pancreatic cancer cells, Bruceine A (Lu et al., 2021) exhibited cytotoxicity to MIA PaCa-2 cells with IC50 values of 0.029 μmol/L. Brusatol exhibited cytotoxicity to PANC-1 and SW1990 cell lines with IC50 values of 0.36 and 0.10 μmol/L, respectively (Zhao et al., 2011a). BD exerted cytotoxic effects on Hs68 cells with IC50 values > 30 μmol/L (Lau et al., 2009) and inhibited proliferation of Capan-2 cells with IC50 values of 1.1 μmol/L (Liu et al., 2012b). Among the above metabolites, Bruceine A showed the strongest cytotoxic effect in the *in vitro* study of pancreatic cancer.

In intestinal cancer cells, Bruceanol D, E, F (Imamura et al., 1993) exhibited cytotoxicity against HCT-8 ileocecal adenocarcinoma with ED50 values of 0.16–0.67 μmol/L. Brusatol, Bruceine B, BD, and Yadanziolide A (Liu et al., 2011) were evaluated for cytotoxicity against SW480 cell lines with IC50 values 0.1–28.5 μmol/L. Yadanziolides T, Yadanziolide B, Bruceine B, D, E, H (Chen et al., 2011; Liu et al., 2012a) exhibited cytotoxicity against HCT-8 with IC50 values of 1.3–6.7 μmol/L. Among the monomers, Bruceine H had the lowest IC50, indicating that its cytotoxicity was the most significant. In the preparation of BJ, Brucea javanica ethanolic extract (BJEE) exhibited cytotoxicity against HCT-116 and HT29 cells with IC50 values of 8.9 ± 1.32 ug/mL (Bagheri et al., 2018a), 48 ± 2.5 ug/mL, respectively (Bagheri et al., 2018b).

In the study of the mechanism of action, Brusatol exerted anti-tumor effects through multiple mechanisms of action in gastrointestinal tumors. Brusatol (Zhou et al., 2021) reversed the Hep-2 cell epithelial-mesenchymal transition (EMT) process, abolishing the Janus kinase 2 (JAK2)/STAT3 signaling pathway in laryngeal cancer cell line. Brusatol (Chen et al., 2021) induced SGC-7901 cell apoptosis in gastric cancer through the PI3K/AKT/NF-κB pathway. In addition, Brusatol (Xiang et al., 2017) induced apoptosis in PANC-1 and PATU-8988 cell lines, possibly through the JNK/p38 mitogen-activated protein kinases (p38 MAPK)/NF-ΚB/STAT3/b-cell lymphoma-2 (BCL-2) pathway. In colorectal cancer, Brusatol functions by facilitating the degradation process of hypoxia-inducible factor-1 (HIF-1α) (Oh et al., 2017), mediated by prolyl hydroxylase (PHD), while concurrently suppressing NRF2 (Evans et al., 2018). Another monomer metabolite with an important role in gastrointestinal tumors is BD. BD (Li et al., 2020) inhibited gastric cancer cell growth and proliferation through the LINC01667/miR-138-5p/Cyclin E1 pathway and induced cell cycle arrest in the s phase of gastric cancer cells. In pancreatic cancer, BD may cause an upregulation of caspase 3, 8, 9, and proapoptotic protein (BAK) protein levels, as well as a downregulation of BCL-2 expression. Furthermore, the p38-MAPK signaling pathway and mitochondrial pathway may also be involved in this process (Lau et al., 2009; Liu et al., 2012b). Furthermore, Bruceine A holds significant relevance in the investigation of digestive tract cancers. Bruceine A (Lu et al., 2021) inhibited pancreatic cancer cell growth by interacting with p38a MAPK P-loop residues. Interestingly, the apparent affinity of P38a MAPK to Bruceine A was higher than that of Brusatol. In the study of preparations, BJOE (Yan et al., 2015) stimulated HCT116 cancer cell apoptosis, which may be related to the reduction of light-chain 3 II (LC3-II), LC3-I expression and inhibition of autophagy. BJEE upregulated BAX, caspase 3, and caspase 9, and activated caspase via extrinsic and intrinsic mitochondria pathways to induce apoptosis (Bagheri et al., 2018b; Bagheri et al., 2018a).

#### 4.2.4 Cancer of the reproductive system

Research on the use of BJ for treating reproductive system cancers has primarily focused on breast cancer, ovarian cancer, and cervical cancer. In the field of research on reproductive system cancer, several metabolites, including Brusatol, Bruceine A, Bruceine B, Bruceantinol, and Brujavanol E, have been investigated for their potential impact on breast cancer. Additionally, Bruceines B, BD, and H have shown significant cytotoxic effects against ovarian cancer. The mechanism of action of BD in breast cancer is Preliminary research has been conducted, and compound preparations mainly involve BJOE.

Brusatol, Bruceine B, BD, Bruceantinol, Bruceine A, Bruceantarin, and Brujavanol E (Liu et al., 2011; Su et al., 2013a; Ye et al., 2015; Chumkaew et al., 2019) demonstrated significant cytotoxicity activity against MCF-7 cell lines with IC50 values of 0.08–10 μmol/L. Brusatol, Bruceantinol, Bruceine A, and Bruceantarin (Ye et al., 2015) demonstrated antineoplastic activity in MDA-MB-231 cells with IC50 values in the ranges of 0.081–0.238 μmol/L. Among the monomers of BJ against breast cancer, Bruceantinol showed the strongest cytotoxic effect. In the extract of BJ, the warmed water extract of BJ (Lau et al., 2005) induced cell death in the MDA-MB231 breast cancer cell line with an IC50 of approximately 50 ug/mL. BJ ethanolic extract (Haryanti et al., 2019) had anti-migratory effects on 4T1 cells at lower doses and exhibited selective cytotoxicity and induction of apoptosis at 90 ug/mL. In ovarian cancer, Bruceosides D, E, and F (Ohnishi et al., 1995) showed selective cytotoxicity in ovarian cancer cell lines, with log GI50 values in the range of −4.44 to −5.72. Javanicolide H, Bruceine B, D, E, H, Dehydrobrusatol, and Javanicolide E (Liu et al., 2012a) exhibited cytotoxicity against SKVO3, having IC50 values in the range 0.12–2.5 μmol/L. In the study of cervical cancer, Quassilactone A and B (Su et al., 2020) exhibited cytotoxic activity against HeLa with IC50 values of 78.95 ± 0.11 and 92.57 ± 0.13 μmol/L.

In the investigation of the mechanism of action, it was observed that BD triggered apoptosis, migration, and invasion of breast cancer cells by suppressing epithelial-mesenchymal transition (EMT) and activating the PI3K/AKT, p38 MAPK, and JNK signaling pathways (Luo et al., 2020; Mohan et al., 2021). Research on the mechanism of BJ’s chemical preparations includes Ethanol Extract of BJ, BJOE, and BJO. Ethanol extract of BJ (Chen et al., 2020a) inhibited autophagy in MDA-MB-231 cells, possibly by activating the PI3K/AKT/mTOR pathway, while being non-toxic in non-target organs. BJOE (Ye et al., 2020) inhibited the expression of oncogene E6 and caused apoptosis in SiHa cells in a way that depended on the dose. This may have happened through the ERK/MAPK and NF-ΚB signaling pathways. BJO (Su et al., 2021) inhibited tumor growth in MDA-MB-231 xenograft mice, modulated the amino acid profile of the host through metabolic changes in the gut microbiota, and then activated mTOR.

#### 4.2.5 Other cancers

Apart from its demonstrated anti-tumor efficacy in the aforementioned tumor types, BJ has also been employed for the therapeutic management of diverse malignancies, including nasopharyngeal cancer, glioma, renal cancer, bladder cancer, and osteosarcoma. Buceoside C, Bruceanol D, E, and F (Fukamiya et al., 1992; Imamura et al., 1993) demonstrated cytotoxic activity against human epidermoid carcinoma of the nasopharynx with ED50 < 0.15 μmol/L. Bruceanol D, E, and F (Imamura et al., 1993) exhibited cytotoxicity against TE-671 medulloblastoma with ED50 values of 0.14–0.22 μmol/L, respectively. Buceoside C, Bruceanol D, E, and F (Fukamiya et al., 1992; Imamura et al., 1993) have shown substantial cytotoxicity against melanoma with ED50 < 0.15 μmol/L. Meanwhile, Bruceoside D, E, and F (Ohnishi et al., 1995) demonstrated selective cytotoxicity in melanoma, with log GI50 values ranging from −4.25 to −4.64. Furthermore, Bruceoside D, E, F (Ohnishi et al., 1995) showed selective cytotoxicity in renal cancer cells with log GI50 values of −4.43 to −4.97. Bruceanol D exhibits the lowest ED50 value, which is measured at 0.14 μmol/L, suggesting a high level of safety.

In the study of anti-tumor mechanisms, Brusatol has been shown to induce apoptosis in head and neck tumors, nasopharyngeal carcinoma, and glioblastoma. Brusatol regulated the expression of key molecular targets (Bcl-xl, Bcl-2, Caspase-9, Caspase-7, Caspase-3, etc.) and proteins, and induced apoptosis through the STAT3 signaling pathway and the Akt/mTOR pathway (Lee et al., 2019; Guo et al., 2020; Dai et al., 2021). Additionally, Brusatol also exerted its inhibitory effect by downregulating the expression of extracellular matrix protein 1 (ECM1) in xenograft tumors of mice (Dai et al., 2021). Another significant metabolite is BD, which has been shown to potentially impede the proliferation and migration of osteosarcoma cells through the inhibition of the STAT3 signaling pathway. It effectively reduces STAT3 phosphorylation in mouse xenograft tumors (Wang et al., 2019). In the study of compound preparations, BJO (Lou et al., 2010) induced apoptosis of T24 bladder cancer cells, possibly by upregulating caspase-3 and caspase-9 expression by activating the caspase pathway and inhibiting the NF-ΚB and Cyclooxygenase-2 (COX-2).

**FIGURE 10:**
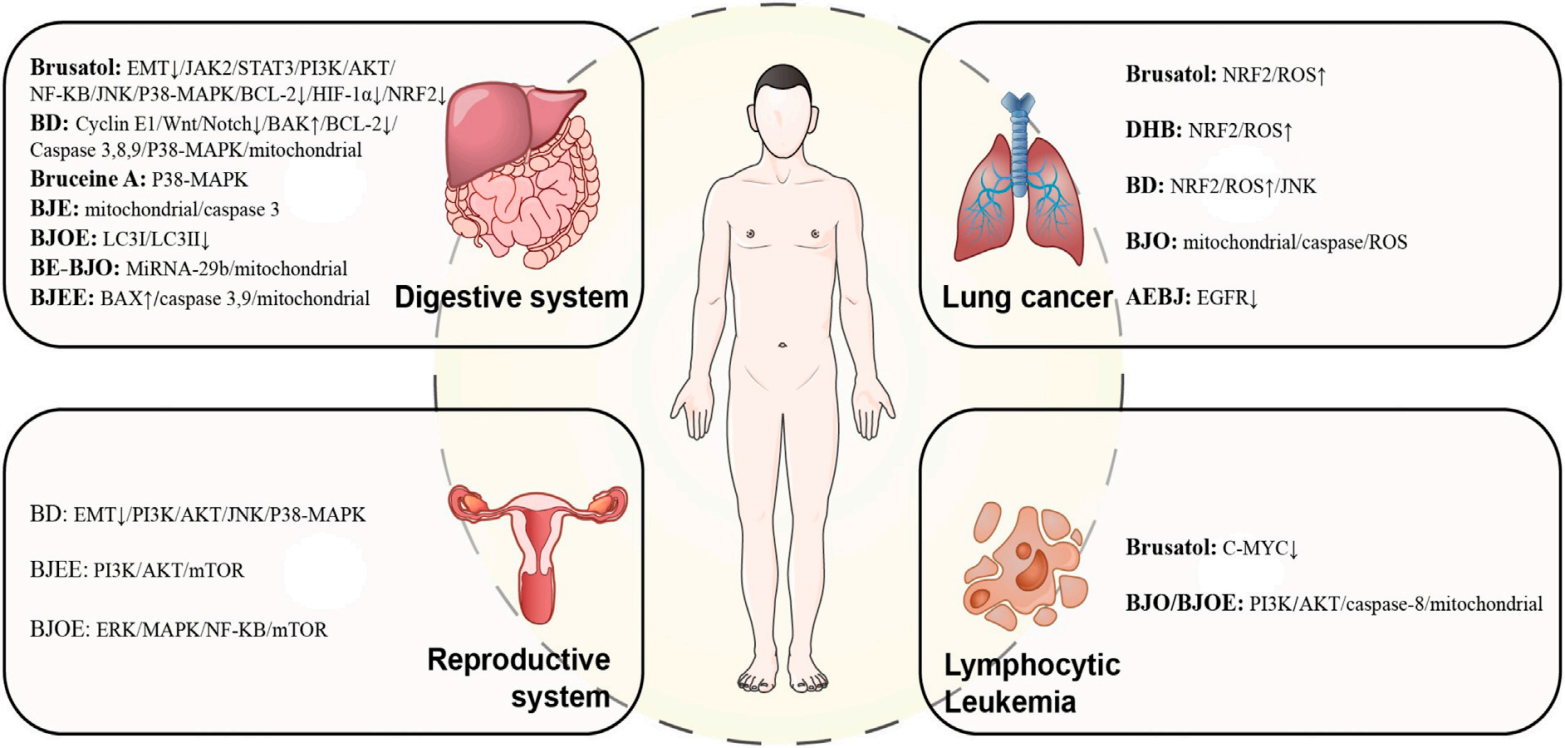
The pharmacological mechanism of BJ’s anti-tumor.

Figure 10 presents an overview of the anti-tumor mechanism of BJ.

### 4.3 Other advances in pharmacological research of BJ

Besides the pharmacological effects listed above, BJ has been shown to have anti-tuberculosis, anti-microbial, anti-muscle atrophy, and lipolytic properties.

DeHydrobrustine, a quassinoid of BJ, has demonstrated inhibitory properties against *mycobacterium tuberculosis* strain H37Rv (Rahman et al., 1997). Research findings indicate that endophytic fungi derived from BJ’s can impede the proliferation of various bacteria, such as *Bacillus subtilis*, *Escherichia coli*, *Staphylococcus aureus*, and *Streptococcus* (Zi-ning et al., 2014). One of the identified peptides, Brucin, which was isolated from BJ, exhibited a notable inhibitory impact on the growth of *Streptococcus* (Sornwatana et al., 2013). Additionally, the BJ extract showed antifungal properties against oral *candida in vitro*, suggesting that it might be helpful as a treatment for fungal infections (Nordin et al., 2013). Brucein A, Brucein B, Brucein C, 3′-hydroxybrucein A, Brusatol, and Bruceantinol (Lahrita et al., 2019) have been reported in the literature to exhibit lipolytic activity. Although the specific mechanism of action is still unclear, these metabolites have shown promising potential in the prevention and treatment of obesity. Moreover, BJ extract and BD can effectively correct splicing errors in surviving motor neuron 2 and alleviate symptoms related to spinal muscular atrophy in mice (Baek et al., 2019).

## 5 Clinical applications of BJ

### 5.1 Progress in preparation of BJ

BJ encompasses a diverse range of metabolites and various formulations. BJ has been commercially available in many forms, such as BJOE injection, BJO oral emulsion, and BJO soft capsules.

BJO preparations have been widely used clinically. However, reports of adverse reactions occur during clinical application, including nausea, vomiting, liver damage, other digestive system damage, itching, rash, skin damage, and nervous system damage such as dizziness and headache (Dan et al., 2017). The adverse reaction to BJO preparation is attributed to the toxicity of its water soluble quassinoids (Rong and Qian, 2010). On the other hand, the primary constituent of BJO injection is oleic acid, which can interact with solvents, compromising its stability and leading to adverse reactions (Lina et al., 2020). Researchers conducted a study on novel formulations to enhance BJO’s stability and mitigate its adverse effects. Through literature review, the preparations of BJ in research include BJ oil-loaded liposomes (Cui et al., 2010), BJO microemulsion (Yang et al., 2014), BJO cationic nanoemulsions (Liu et al., 2016), Luteinizing hormone releasing hormone receptor-targeted BJO liposomes (Ye et al., 2016), brusatol self-microemulsifying drug delivery system (Zhou et al., 2017), Self-assembled stable sponge-type nano-drug delivery system with BJO (Zou et al., 2017), self-nanoemulsifying drug delivery system of BD (Dou et al., 2018), self-microemulsifying drug delivery system (S-SMEDDS) of BJO (Huang et al., 2018), Disulfide-linked Pluronic-linoleic acid composed of redox-sensitive micelles of brusatol (Zhang et al., 2018a), BJO gastroretentive floating bead (Zhang et al., 2018b), Glycosaminoglycan-placental chondroitin sulfate-modified nanoparticle delivery brusatol system (Chen et al., 2020b), 3-β-homoalanine conjugate of brusatol (Hwang et al., 2020). These new preparations showed higher anti-tumor activity, better bioavailability, and lower toxicity to animals in the study.

### 5.2 Traditional clinical application of BJ

The existing body of literature predominantly centers around the traditional clinical application of BJ in treating dysentery, cutaneous warts, and corn. For more comprehensive information regarding the pertinent clinical studies, please consult Table 9.

**TABLE 9:** Traditional clinical application of BJ.

Clinical application	Dosage form	Number of subjects	Result	Ref.
Dysentery	BJ capsule	12 cases	Effective rate: 91.7%	Qiang (1999)
Genital warts	BJ compound lotion	72 cases	Effective rate: 93.1%	Caiyong et al. (2019)
	BJ extract	34 cases	Effective rate: 94.1%	Jianmin et al. (2010)
	BJ extract	68 cases	Effective rate: 97.0%	Liwen and Wenlei (2016)
			HPV clearance rate: 55.9%	
	BJ compound lotion	63 cases	Effectiverate: 100%	Caiyong et al. (2009)
	BJ Cream	165 cases	Effective rate: 100%	Jumin (2006)
	BJ extract	60 cases	Effective rate: 92.3% (Warts < 2 mm)	Gangsheng et al. (2003)
			72.3% (2–5 mm)	
Flat wart	BJ tincture	260 cases	Effective rate: 93.4%	Ge (2001)
	BJ extract	46 cases	Effective rate: 82.6%	Hong (2007)
Plantar warts	BJ patch	60 cases	Effective rate: 88.3%	Fanxiu (2014)
	BJ Cream	60 cases	Effective rate: 90.8%	Hengan et al. (2008)
Corn	BJ extract	120 cases	Effective rate: 77.5%	Jianming (2004)
	BJ poultice	36 cases	Effective rate: 91.7%	Jianjun et al. (2000)

### 5.3 Antitumor clinical application of BJ

#### 5.3.1 BJ combined with radiotherapy and chemotherapy

Chemotherapy and radiation, as traditional cancer treatment methods, often come with severe side effects, including myelosuppression, mucositis, and hair loss (Cao et al., 2014). The advancement of molecular biotechnology has brought about targeted drugs that have proven to be beneficial for cancer patients. However, these drugs still face significant challenges, such as drug resistance and side effects, limiting their clinical application (Wu et al., 2022b). The combined use of BJ with radiotherapy and chemotherapy has been shown to enhance antitumor activity while minimizing cytotoxicity and toxic side effects. BJ is presently employed as adjunctive therapy for neoplasms and is anticipated to assume a more prominent function in the domain of anti-neoplastic interventions.

Cancers of the lungs, liver, stomach, pancreas, esophageal, and colon are the main types of cancer that use the BJ and chemotherapy medication combination. Out of these choices, combining BJOE with chemotherapy drugs has been the subject of a lot of research. It has shown impressive therapeutic benefits, such as better quality of life, more effective treatment, and fewer adverse events. The complex composition of BJOE poses a significant challenge to studying its mechanism of action in combination with chemotherapy drugs for malignancies. Therefore, using monomeric metabolites in BJ in combination with chemotherapy drugs for anti-tumor use has become a promising breakthrough. In lung cancer, dehydrobruceine B sensitizes A549 cells to cisplatin by regulating mitochondrial apoptosis, thereby reducing their resistance to cisplatin (Huang et al., 2017b). Canthin-6-one, when combined with cisplatin, induces cytotoxicity and has the potential for anti-drug resistance (Samat et al., 2017). It is noteworthy that the combination of Brusatol and chemotherapy has shown a significant synergistic anti-tumor effect in colorectal cancer, pancreatic cancer, and pituitary tumors (Lu et al., 2017; Chen et al., 2018a; Wu et al., 2021). At the same time, research has found that brusatol can overcome the chemical resistance of a wide range of cancer types (Harder et al., 2017; Chen et al., 2018a). Based on the findings above, investigating the potential of combining brusatol with chemotherapeutic medications holds promise as a prospective avenue for future research. The integration of this combination has the potential to enhance therapeutic efficacy and present a novel therapeutic approach for individuals with drug-resistant tumors. Specific information on clinical studies is shown in Table 10.

**TABLE 10:** BJ combined with radiotherapy and chemotherapy.

Metabolites of BJ	Combination	Type of cancer	Mechanism/Advantage	Ref.
BJO	Radiotherapy	Esophageal cancer/ECA109 cells	Hypoxia-inducible factors 1α ↓	Pan et al. (2018)
BJOE	Radiotherapy	Esophageal cancer/EC109 cells	Cyclin D1-CDK4/6 axis	Qiu et al. (2019)
BJOE	Radiotherapy	Placental villous Carcinoma/JAR cells	Cyclin D1-CDK4/6 axis	Qiu et al. (2019)
BJO	Radiotherapy	Esophageal cancer	CRR ↑ RR ↑	Shan et al. (2011)
		80 patients	Karnofsky score ↑	
BJO	Radiotherapy	Esophageal cancer	CRR ↑ PRR ↑	Wang et al. (2017)
		1,269 patients	Quality of life ↑	
BJOE	Radiotherapy	Esophageal cancer	HIF-1α ↓	Pan et al. (2018)
		/20 patients	Apoptotic rate ↑	
BJO	Gemcitabine	Pancreatic cancer/mouse model	Survival rate ↑	Yang et al. (2020)
			Tumor growth rate ↓	
BJOE	Manganese	Endometrial cancer/RL95-2 cells	Proliferation ↓	Hu et al. (2021)
			Apoptosis ↑	
BJOE	Transarterial chemoembolization (TACE)	Liver cancer/64 patients	RR ↑	JIN et al. (2015)
BJOE	Oxaliplatin, Irinotecan, Paclitaxel, Docetaxel, Fluorouracil, etc.	Gastric cancer/75 patients	Karnofsky score ↑	Liu et al. (2013)
			Quality of life ↑	
BJ emulsion	FOLFOX4	Gastric cancer/150 patients	Vascular Endothelial Growth Factor ↓	Chen et al. (2018b)
			Recurrence rate ↓	
BJOE	Oxaliplatin, Cisplatin paclitaxel, etc.	Gastric cancer/912 patients	RR ↑ PS ↑	Wu et al. (2018)
			ADR ↓	
BJOE	CapeOX chemotherapy	Rectal cancer/80 patients	Relieve nausea	Meng et al. (2021)
BJOE	PP chemotherapy	Lung cancer/58 patients	Quality of life ↑ ORR ↑	Lu et al. (2013)
			Incidence of leukopenia ↓	
BJO	Doxorubicin	Breast cancer/MCF-7 cells	Apoptosis ↑	Li et al. (2019b)
			Resistance ↓	
Dehydrobruceine B	Cisplatin	Non-small-cell Lung carcinoma/A549 cells	Regulation of mitochonDrial apoptosis pathway	Huang et al. (2017b)
			Resistance ↓	
Canthin-6-one	Cisplatin	Prostate/PC3 cells	Block G 2/M cancer cells	Samat et al. (2017)
		Cervical cancer/HeLa cells	Resistance ↓	
Brusatol	Cisplatin	Colon cancer/CT-26 cells	Procaspase-3/9 ↓	Chen et al. (2018a)
			Cytochrome c/BCL-2 ↑	
Brusatol	Gemcitabine/5-fluorouracil	Pancreatic cancer	Inhibit the activation of NF-κB	Lu et al. (2017)
		/PANC-1 cells		
Brusatol	Gemcitabine/5-fluorouracil	Pancreatic cancer/mouse model	E-cadherin expression ↑	Lu et al. (2017)
			Resistance ↓	
Brusatol	Cabergoline	Pituitary adenoma/GH3/MMQ cells mouse model	ROS ↑	Wu et al. (2021)
			Inhibit the mTORC1 signaling pathway	
Brusatol	Metformin	Endometrial cancer cells	Nrf2/AKR1C1 ↓	Wang et al. (2016b)
			Progesterone resistance ↓	
Brusatol	Cisplatin/Desferal	Ovarian cancer cells	Nrf2 ↓ SLC40A1 ↑	Wu et al. (2017)
			Resistance ↓	
Brusatol	Oxaliplatin	Colorectal cancer LS174T cells	Nrf2 ↓ P-glycoprotei ↑	Mostafazadeh et al. (2022)
			Resistance ↓	

CRR, Complete Response rate; PRR, partial response; RR, Response rate; PS, performance status; ADR, Adverse reactions; ORR, overall response rate; ↑: rise ↓: decline.

#### 5.3.2 BJ combined with other drugs

Several studies have shown that the co-administration of BJ with paclitaxel, cantharidin, baicalin, and other traditional Chinese medicines exhibits a synergistic anti-tumor effect (Ma et al., 2013; Ji et al., 2014; Chen et al., 2015). Furthermore, combining BJ with targeted drugs for cancer treatment has also shown promising results in synergistic anti-tumor effects and combating drug resistance in tumor cells (Peng et al., 2021; Wu et al., 2022a). In addition to the anti-tumor field, research has shown the efficacy of BJ combined with psoralen in treating Pneumocystis carinii infection (Qin et al., 2006; Dai et al., 2007). Clinical studies on BJ combined with other drugs to treat diseases are shown in Table 11.

**TABLE 11:** BJ combined with botanical drug and targeted drug.

Metabolites of BJ	Combination	Type	The advantage of combination	Ref.
BJO	Docetaxel	Rat Model	AUC ↑ T1/2 ↑	Ma et al. (2013)
BJ	Cantharidin	Clinical study	Quality of life ↑	Ji et al. (2014)
BJO	Baicalin	Cell experiment/A549 cells	Apoptosis ↑	Chen et al. (2015)
BJO	Anlotinib	Mouse Model/Small-cell lung cancer	Liver metastases ↓	Peng et al. (2021)
			Angiogenesis ↓ weight loss ↓	
Bruceine H	Gefitinib	Xenografted tumors in nude mice	PC-9/GR ↓	Wu et al. (2022a)
			NOTCH3 ↓	
Brusatol	Paclitaxel	Breast cancer/MCF-7 cells	Nrf2 ↓ ROS ↑	Wu et al. (2015)
		MDA-MB-231 cells	Resistance ↓	
BD	Sorafenib	Hepatocellular carcinoma/Huh7, Hep3B cells/Mice	Apoptosis ↑	Cheng et al. (2017)
			Tumor growth ↓	
			Jagged1 expression ↓	
BJOE	Psoralea corylifolia	Pneumocystis carinii disease rats	Immunomodulation ↑	Qin et al. (2006)
			Pneumocystis carinii ↓	
BJOE	Psoralea corylifolia	Pneumocystis carinii disease rats	IL-2 ↑ NK cell activity ↑	Dai et al. (2007)
			Immunomodulation ↑	

AUC, Area under the curve.

It is noteworthy that the concurrent administration of BJ and other medications has demonstrated favorable clinical outcomes in various clinical settings. However, the reference value of these clinical studies is limited due to several factors, including small sample sizes, utilization of retrospective data, and inconsistent evaluation of clinical indicators. Another crucial aspect to consider is the inclusion of BJ in clinical studies alongside other medications, which frequently yields favorable clinical outcomes. These outcomes encompass the reduction of toxic side effects and the enhancement of drug efficacy. However, the potential adverse impacts in clinical practice are seldom demonstrated in clinical studies. Therefore, it is imperative to identify and select valuable combination regimens through rigorous clinical studies. Designing high-quality, large-sample, and authoritative clinical studies incorporating comprehensive evaluation indicators is crucial.

## 6 Discussion

In the research on the traditional pharmacological effects of BJ, preliminary studies have been conducted to investigate its pharmacological action mechanism in relation to anti-dysentery and treatment of skin warts and corns. BJ resists dysentery through the NF-κB and Nrf2 signaling pathways (Li et al., 2018; Zhou et al., 2018; Zheng et al., 2021)[25] and is used to treat genital warts by regulating the expression of TLR4 receptors (Weiqi et al., 2023). The research progress on the traditional pharmacological mechanism of BJ has not been addressed in other review literature. This article summarizes and elaborates on the traditional pharmacological mechanism of BJ to provide reference information for further research in this field. BJ’s current research primarily focuses on the anti-tumor properties. In China, BJO and BJOE have been approved for the treatment of lung cancer, brain metastasis of lung cancer, and cancers affecting the digestive tract. The studies conducted by BJO and BJOE have demonstrated cytotoxic properties against various types of tumors (Zhang et al., 2011; Shi et al., 2015; Yan et al., 2015; Wang et al., 2016a). In the study of the mechanism of action, BJ mainly plays a role in lymphocytic leukemia through the PI3K/Akt signaling pathway (Zhang et al., 2011; Zhang et al., 2021), lung cancer through the NRF2 (Zhao et al., 2016; Xie et al., 2021) and JNK (Yang et al., 2013) signaling pathways. The Wnt/Notch (Cheng et al., 2017) and PI3K/Akt/mTOR (Ye et al., 2018) signaling pathways are involved in liver cancer. It also plays a role in laryngeal cancer through the JAK2/STAT3 signaling pathway (Zhou et al., 2021). The p38 MAPK signaling pathway (Lau et al., 2009; Liu et al., 2012b; Xiang et al., 2017) has been found to play a role in lymphoma and gastrointestinal cancer. A role in breast cancer is played by the PI3K/AKT, p38 MAPK, and JNK signaling pathways (Luo et al., 2020; Mohan et al., 2021). Existing studies have shown that BJ exerts pharmacological effects through multiple signaling pathways. It should be noted that the current research only extensively explores the signaling pathways of pharmacological effects and does not indicate which signaling pathways are primarily involved in different pharmacological effects. At the same time, the effects of BJ on genes and proteins associated with the upstream and downstream signaling pathways have not been thoroughly studied. Research on the pharmacological mechanism of BJ lacks a complete signaling pathway system as theoretical support.

BJO and BJOE have been widely used in clinical practice. Nevertheless, numerous instances of adverse responses have been observed during the clinical implementation, encompassing manifestations such as rash, vomiting, dizziness, palpitations, and various other untoward effects (Dan et al., 2017). About medicinal aspects, BJ possesses a diverse array of metabolites and exhibits inherent toxicity attributed to its water-soluble quassinoid chemicals. At the same time, excipients such as soy lecithin and glycerol are added, and the purity and stability of BJO and BJOE are easily changed during the production, storage, and use processes, leading to adverse drug reactions (ADR). Due to the complexity of BJO and BJOE formulas and the occurrence of adverse reactions, the widespread application of BJ worldwide is limited. Another issue that requires attention with BJO and BJOE is the addition of multiple excipients, making it challenging to identify adulteration of the preparations. Huang et al. used direct injection-electrospray ionization ultra-high resolution mass spectrometry technology to identify 69 metabolites in BJO for adulteration identification of BJO, providing theoretical support for the quality standards of BJO (Yan et al., 2023). Limited by the aforementioned factors, the clinical application of BJO and BJOE has not been able to progress towards broader implementation. At present, there have been many studies on the improvement of preparations (Cui et al., 2010; Yang et al., 2014; Liu et al., 2016). However, these new preparations are limited by factors such as clinical efficacy and drug economy. They still require a significant number of high-quality studies to be conducted to verify their suitability for clinical applications.

It is precisely because of the complexity of the metabolites of BJO and BJOE that studying the pharmacological mechanism of action becomes difficult. Studying the pharmacological mechanism of the metabolites of BJ monomer may lead to future breakthroughs. Among the monomer metabolites of BJ, we screened out 15 metabolites with research potential. In traditional pharmacological research of BJ, five monomer metabolites exhibit strong pharmacological activity. The monomeric metabolites include Brusatol, Bruceine A, and Bruceantinol, which have anti-dysenteric effects (Subeki et al., 2007; Zhou et al., 2018). Bruceantin has shown the most potent anti-malarial effect (Guo et al., 2005). BD and brusatol are potentially effective in treating warts and corn (Jingli et al., 2017; Weiqi et al., 2023), and the hypoglycemic effect of BD and Bruceine E warrants further research (NoorShahida et al., 2009). Modern research on BJ mainly focuses on its pharmacological effects in treating tumors. *In vitro* cell line studies, 14 monomeric metabolites exhibited significant cytotoxic effects. Bruceanol D, Brusatol, and Bruceantin exhibit strong cytotoxic effects in lymphocyte cell line studies (Imamura et al., 1993; Mata-Greenwood et al., 2002; Liu et al., 2011). Brusatol, Bruceloside C, and BD have demonstrated potent anti-tumor effects in lung cancer cell line studies (Fukamiya et al., 1992; Liu et al., 2011; Tan et al., 2019). Brusatol, BD, and Bruceine B exhibit significant cytotoxic effects on liver cancer cell lines (Liu et al., 2011; Liu et al., 2012a). Bruceanol G exhibits potent cytotoxicity in oral cancer (Imamura et al., 1995), while Brusatol is effective against laryngeal cancer (Zhou et al., 2021). BD and Javanicolide H exhibit potent cytotoxic effects in gastric cancer (Chen et al., 2011; Liu et al., 2012a), while BD, busatol and Bruceine H demonstrate efficacy in colorectal cancer (Chen et al., 2011; Liu et al., 2011). Bruceine A and brusatol showed the strongest cytotoxic effect in pancreatic cancer (Zhao et al., 2011a; Lu et al., 2021). Brusatol, Bruceine A, Bruceine B, Bruceantinol, and Brujavanol E exhibit cytotoxic solid effects against breast cancer (Okano et al., 1985; Su et al., 2013a; Ye et al., 2015; Chumkaew et al., 2019). Bruceine B, BD, and Bruceine H exhibit strong cytotoxic effects on ovarian cancer (Liu et al., 2012a). Bruceanol D, E, and F exhibit strong cytotoxic effects on nasopharyngeal squamous cell carcinoma, melanoma, and medulloblastoma (Lee et al., 1977; Fukamiya et al., 1992; Imamura et al., 1993). Through the study of BJ monomer metabolites, researchers screen out metabolites with research potential and explore the possibility of applying these compounds in clinical practice in the future.

The different metabolites of BJ possess diverse pharmacological properties. However, both the traditional pharmacological effects of BJ and the related mechanisms of modern pharmacological applications are still in the preliminary exploration stage. A comprehensive gene signaling pathway has not yet been established to elucidate BJ’s pharmacological effects fully. Secondly, BJO and BJOE have been utilized in clinical applications. However, it is essential to note that adverse reactions may occur due to drug and preparation factors during clinical application. Finally, BJO and BJOE have different metabolites, making it quite difficult to explore their exact pharmacological mechanisms of action. Exploring the monomer metabolites for clinical practice could be a significant breakthrough.

## 7 Conclusion

In summary, the existing evidence regarding the safety and effectiveness of BJO and BJOE in clinical applications is confined mainly to clinical observational studies and needs robust evidence-based support. In terms of researching the mechanism of action, it is very challenging to study the mechanism of action of BJO and BJOE due to their multiple ingredients. There have been reports of adverse reactions in the clinical use of BJO and BJOE. The factors above constrain the global promotion of BJ. We identified 15 metabolites with research potential in BJ and aim to investigate further the potential application of these metabolites in clinical practice in the future. We suggest that future research should focus on three main areas: integrating the pharmacological mechanisms of BJ, establishing a comprehensive gene signaling pathway system, and providing theoretical support for clinical applications. The second approach is to improve the preparations of BJ by selecting safer and more soluble excipients, enhancing the stability of BJO and BJOE, and reducing the occurrence of adverse reactions. The third step is to screen monomer metabolites with research potential for further investigation and to explore their potential for clinical application.

Although BJ currently faces many challenges in more comprehensive clinical implementation, the continuous deepening of research in this field and the application of new technologies such as molecular docking technology, high-throughput screening technology, and cell membrane chromatography technology will undoubtedly accelerate the development of BJ. BJ will have broader clinical applications worldwide.

## Figures and Tables

**Table:** 

**TCM**	Traditional Chinese Medicine
**CBD**	Chinese botanical drugs
**BJ**	Brucea javanica
**NRF2**	nuclear factor erythroid-2 related factor 2
**JNK**	Jun n-terminal kinase
**STAT3**	signal transducer and activator of transcription 3
**NF-κB**	nuclear factor kappa-B
**RhoA/ROCK**	member A/rho-associated kinase
**BJO**	Brucea javanica oil
**BJOE**	Brucea javanica oil emulsion
**TLR4**	toll-like receptor 4
**HPV**	human papillomavirus
**DNA**	Deoxyribonucleic acid
**BD**	Bruceine D
**EAF**	ethyl acetate fraction
**BJE**	Ethyl acetate Extract of BJ
**ILS**	the ratio of mean murine life span in the experimental group to murine in the control group
**c-MYC**	cellular-myelocytomatosis viral oncogene
**PI3K**	Phosphoinositide 3-kinase
**Akt**	Protein kinase B
**EGFR**	epidermal growth factor receptor
**DHB**	Dehydrobruceine B
**ROS**	Reactive Oxygen Species
**NSCLC**	non-small-cell lung carcinoma
**AE-BJ**	Aqueous BJ extract
**HCC**	Hepatocellular -carcinoma
**mTOR**	mammalian target of rapamycin
**EMT**	epithelial-mesenchymal transition
**JAK2**	janus kinase 2
**p38 MAPK**	p38 mitogen-activated protein kinases
**BCL-2**	b-cell lymphoma-2
**PHD**	prolyl hydroxylases
**HIF-1α**	hypoxia-inducible factor-1
**BAK**	proapoptotic protein
**ERK**	extracellular regulated protein kinases
**MAPK**	mitogen-activated protein kinase
**ECM1**	extracellular matrix protein 1
**LC90**	Lethal Concentration 90
**MIC**	minimum inhibitory concentration
**LC3-II**	light-chain 3 II
**BJEE**	Brucea javanica ethanolic extract
**COX-2**	Cyclooxygenase-2
**S-SMEDDS**	self-micro emulsifying drug delivery system
**CRR**	Complete Response rate
**PRR**	partial response
**RR**	Response rate
**PS**	performance status
**ADR**	Adverse reactions
**ORR**	overall response rate
**AKR1C1**	Aldo-keto reductase family 1 member C1
**SLC40A1**	Solute Carrier Family 40 Member 1
